# Inner ear pathologies impair sodium-regulated ion transport in Meniere’s disease

**DOI:** 10.1007/s00401-018-1927-7

**Published:** 2018-11-02

**Authors:** Andreas H. Eckhard, MengYu Zhu, Jennifer T. O’Malley, Gordon H. Williams, Johannes Loffing, Steven D. Rauch, Joe B. Nadol, M. Charles Liberman, Joe C. Adams

**Affiliations:** 1 Otopathology Laboratory, Massachusetts Eye and Ear Infirmary, Boston, MA USA; 2 Division of Endocrinology, Diabetes, and Hypertension, Department of Medicine, Brigham and Women’s Hospital, Harvard Medical School, Boston, MA USA; 3 Institute of Anatomy, University of Zurich, Zurich, Switzerland; 4 Eaton-Peabody Laboratories, Massachusetts Eye and Ear Infirmary, Boston, MA USA; 5 Department of Otolaryngology, Harvard Medical School, Boston, MA USA; 6 Vestibular Division, Department of Otolaryngology, Massachusetts Eye and Ear Infirmary, Boston, USA; 7 Massachusetts General Hospital, Boston, MA USA; 8 Present Address: Department of Otorhinolaryngology, University Hospital Zurich, Frauenklinikstrasse 24, 8091 Zurich, Switzerland

**Keywords:** Meniere’s disease, Endolymphatic sac, Endolymphatic hydrops, Sodium, Aldosterone

## Abstract

Meniere’s disease (MD), a syndromal inner ear disease, is commonly associated with a pathological accumulation of endolymphatic fluid in the inner ear, termed “idiopathic” endolymphatic hydrops (iEH). Although numerous precipitating/exacerbating factors have been proposed for MD, its etiology remains elusive. Here, using immunohistochemistry and in situ protein–protein interaction detection assays, we demonstrate mineralocorticoid-controlled sodium transport mechanisms in the epithelium of the extraosseous portion of the endolymphatic sac (eES) in the murine and human inner ears. Histological analysis of the eES in an extensive series of human temporal bones consistently revealed pathological changes in the eES in cases with iEH and a clinical history of MD, but no such changes were found in cases with “secondary” EH due to other otological diseases or in healthy controls. Notably, two etiologically different pathologies—degeneration and developmental hypoplasia—that selectively affect the eES in MD were distinguished. Clinical records from MD cases with degenerative and hypoplastic eES pathology revealed distinct intergroup differences in clinical disease presentation. Overall, we have identified for the first time two inner ear pathologies that are consistently present in MD and can be directly linked to the pathogenesis of EH, and which potentially affect the phenotypical presentation of MD.

**Electronic supplementary material:**

The online version of this article (10.1007/s00401-018-1927-7) contains supplementary material, which is available to authorized users.

## Introduction

Meniere’s disease (MD) [36] is a syndrome that affects the inner ear. MD is defined and diagnosed based on recurrent fluctuant vestibular (rotational vertigo) and auditory (hearing loss, tinnitus, aural fullness) symptoms [4, 31]. Although MD is generally acknowledged as a definable clinical entity, it remains unclear whether only one etiopathology exists or whether multiple different pathologies can elicit the characteristic symptoms. The latter is suggested by various observations: (1) many precipitating and exacerbating factors have been associated with MD [39, 45]; (2) the frequency, duration and severity of symptoms are highly variable within and between MD patients [17], (3) other disorders can present with similar symptoms [19, 21], (4) the overaccumulation of endolymphatic fluid in the inner ear, i.e., (idiopathic) endolymphatic hydrops (EH), long considered the underlying pathology and cause of MD [52, 22, 20, 41, 32], has been observed in patients without MD symptoms [46, 33] and in cases of other otological diseases (secondary EH; [46, 33] or no otological disease (asymptomatic EH; [37, 43]; and (5) despite many histopathological studies (reviewed in [48, 34]), no distinctive cellular or molecular pathology has been consistently linked to MD.

Nevertheless, several experimental and clinical observations implicate the inner ear’s endolymphatic sac (ES) and endolymphatic Na^+^ balance in the pathogenesis of idiopathic EH and MD: (1) destruction of the ES in animal models leads to EH [26], (2) epithelia lining the parts of the endolymphatic spaces exhibit Na^+^ transport capacity [25], (3) aldosterone (ALDO)—the major hormonal regulator of salt and water balance—exacerbates EH in animal models (reviewed in [47]), (4) high Na^+^ intake can trigger MD attacks [10], and (5) MD patients on a low- or stable-salt diet have decreased symptom severity [45, 18].

**Fig. 1:**
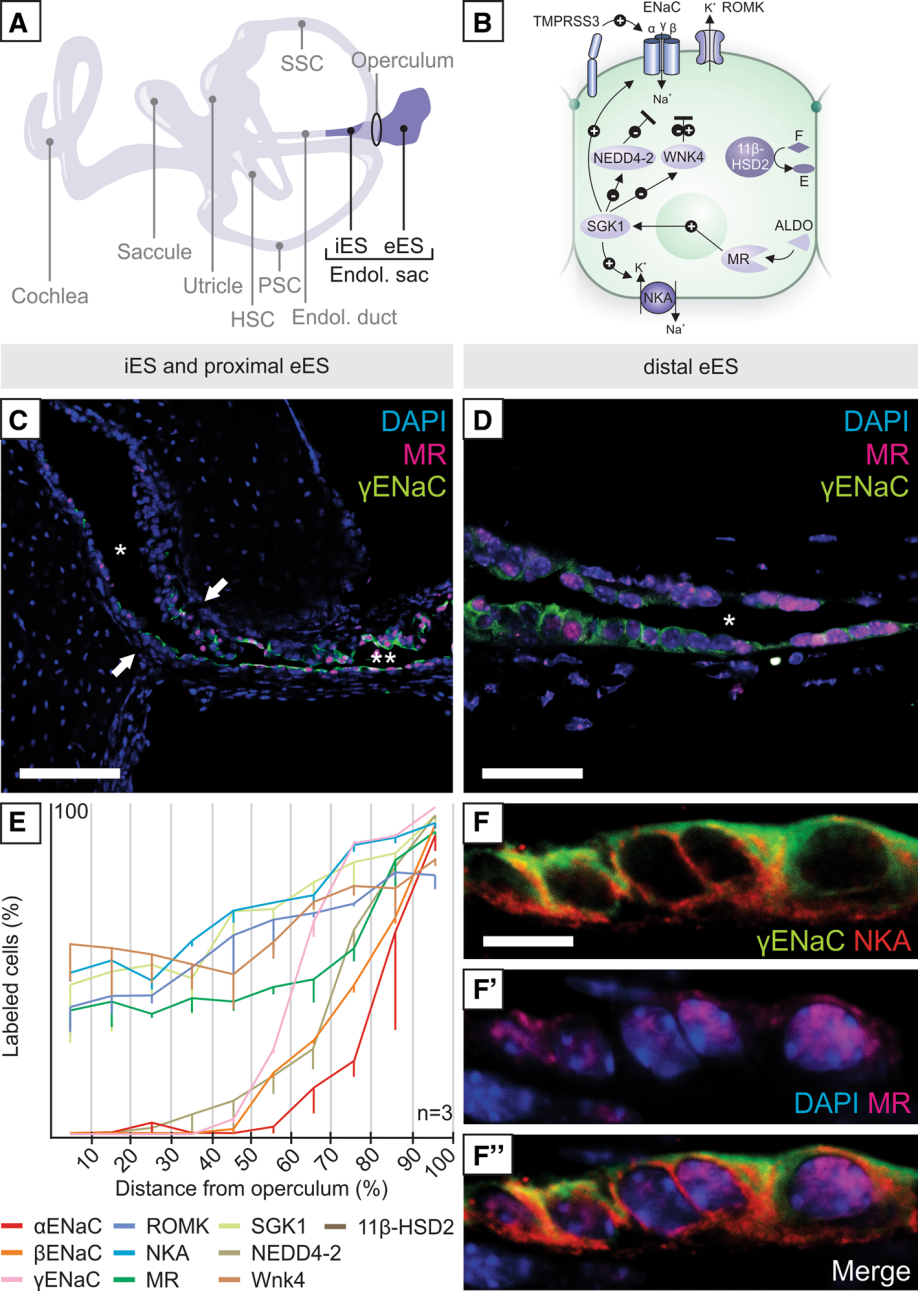
Immunolocalization of aldosterone (ALDO)-regulated ion transport proteins in the murine endolymphatic sac (ES). **a** Schematic of the murine inner ear with the intraosseous (iES) and extraosseous (eES) portions of the endolymphatic sac highlighted. **b** Schematic of ALDO regulation as studied in the kidney’s aldosterone-sensitive distal nephron (refer to results section for details on molecular interactions). **c**, **d** Immunostaining for γENaC and MR shows minimal labeling in the iES (lumen marked with * in **c**), increased labeling in the proximal eES (lumen marked with ** in **c**) with an abrupt onset in the operculum (arrows in **c**), and strong labeling in the distal eES (lumen marked with * in **d**). **e** Proximal-to-distal gradient of ALDO-regulated proteins in the eES. Quantification was performed by dividing the eES into ten equal segments and counting labeled cells in each segment (means and standard deviations; *n* = 3). **f**–**f′** Confocal images demonstrating the polarized localization of γENaC (apical membranes), NKA (basolateral membranes), and MR (nuclei) in the eES. Scale bars: **c** 100 µm; **d** 50 µm; **f**–**f**″ 20 µm

The ES is a nonsensory epithelial appendage of the membranous labyrinth of the inner ear (Fig. 1a). In this study, we used immunohistochemistry and proximity ligation assays to map ALDO-regulated Na^+^ transport proteins in the ES in normal and salt-challenged mice and in normal and MD-affected humans. The channel/transport proteins and ALDO-related signaling molecules we found in the ES are similar to those in the ALDO-sensitive distal nephron, where highly regulated, ALDO-dependent Na^+^ reabsorption is carried out to maintain whole-body sodium and volume homeostasis (reviewed in [30]).

In the murine ES, these ALDO-regulated Na^+^ channels/transporters were responsive to changes in salt intake as seen in the kidney epithelia. In inner ears from patients with MD and idiopathic EH, we found consistent ES abnormalities, i.e., either epithelial degeneration or developmental hypoplasia. Retrospective chart review indicated phenotypic differences between cases with degenerative and hypoplastic ES pathology, with respect to disease laterality, age of onset, comorbidities, and family history. Together, our results strongly implicate the extraosseous portion of the ES and disruptions in the ALDO-sensitive Na^+^ transport cascade it expresses in the generation of EH and MD.

## Materials and methods

### Animals

Male mice of the CBA/CaJ strain were purchased from the Jackson Laboratory (Bar Harbor, ME) and were used in this study between 6 and 8 weeks of age. The animals were kept in an in-house animal facility with a uniform diurnal lighting cycle (12 h/12 h) and free access to food and water.

### Sodium diets and metabolic balance studies

Mice were kept for 7 days on a purified AIN-93 M maintenance diet (TestDiet, St. Louis, MO) with either a standard Na^+^ content (0.14% Na^+^), a low Na^+^ content (0.04% Na^+^), or a high Na^+^ content (4.00% Na^+^), similar to previously established protocols [29]. Animals on standard-Na^+^ and low-Na^+^ diets received tap drinking water, while those on the high-Na^+^ diet had access to 0.9% saline. On the last day of the dietary cycle, all animals were housed individually in metabolic cages. During this 24-h period, fluid intake and urinary output was measured, and 24-h urine samples were collected.

### Plasma and urine analysis

After 7 days on a standard-Na^+^ diet, a low-Na^+^ diet, or a high-Na^+^ diet, mice were killed with an intraperitoneal (i.p.) injection of sodium pentobarbital (100 mg/kg), and cardiac blood samples were collected. All blood samples were collected between 7.30 AM and 8.30 AM, when endogenous plasma levels of ALDO in mice reach their diurnal low [11]. The blood samples were centrifuged, and the plasma was collected in sterile tubes. Urine samples were treated with 1% boric acid. All plasma and urine samples were frozen and kept at − 80 °C. Plasma/urine ALDO levels were determined using an enzyme-linked immunosorbent assay (ELISA; IBL International, Hamburg, Germany) with very low cross-reactivity to corticosterone (> 0.003%, according to the manufacturer).

### Animal tissue processing

All animals were killed (sodium pentobarbital (100 mg/kg), i.p.) prior to blood and tissue (temporal bone [TB], kidney) sampling. All tissues that were collected for immunohistochemical analyses were fixed (fixatives are listed in Supplementary Table 1) overnight (ON) at room temperature (RT). The TBs were decalcified in 0.12 M ethylenediaminetetraacetic acid (EDTA) for 1 week at RT. Then, the tissues were dehydrated in an ascending ethanol series, cleared with xylenes (Sigma, St. Louis, MO), and incubated in melted paraffin ON. The blocks were solidified and sliced into 10 μM sections using a rotary microtome (Reichert 2030 Microtome, Bensheim, Germany); the sections were mounted on precoated glass slides (Superfrost™ Plus, Thermo Fisher, Pittsburgh, PA) and stored at RT.

### Diagnostic criteria and nomenclature applied to human cases

We classified archival TB samples from humans with EH based primarily on the etiology of EH, which can be either secondary or idiopathic. By definition, secondary EH is associated with a history of certain otological or systemic diseases that are believed to cause secondary EH [13], although the detailed pathophysiological connection between the diseases and secondary EH is mostly unknown. In contrast to secondary EH, idiopathic EH, by definition, occurs spontaneously and has unknown etiology [27]. In the present study, patients were classified as having secondary EH when their clinical records mentioned one or more of the diseases that supposedly cause secondary EH (Supplementary Table 2), and patients were classified as having idiopathic EH when their medical history was negative for those diseases. Next, patients with idiopathic EH were classified as having MD when their history of otological symptoms matched the diagnostic criteria for “definite” MD [4]. Patients with secondary EH were classified as having Meniere’s symptom complex when their clinical records fulfilled the diagnostic criteria for “definite” MD [4]. All individuals without a clinical history of MD or Meniere’s symptom complex, respectively, were classified as having had other otological (non-Meniere’s-like) symptoms or no otological symptoms (see also Table 1).

**Table 1:** Frequency of eES pathologies and clinical Meniere’s/non-Meniere’s symptoms

	Otological diagnosis	ES pathology		
		Degeneration	Hypoplasia	None
Idiopathic endolymphatic hydrops, *n* = 24 (42)	Meniere’s disease	13 (25)	9 (11)	1 (2) (3^a^)
	Non-Meniere’s otological symptoms	1 (1)	0	0
	none	0	0	0
Secondary endolymphatic hydrops, *n* = 39 (58)	Meniere’s symptom complex	0	0	3 (5) (1^a^)
	Non-Meniere’s otological symptoms	1 (2)	0	30 (46)
	none	0	0	1 (1) (3^b^)
Normal controls, *n* = 10 (20)	Meniere’s symptom complex	0	0	0
	Non-Meniere’s otological symptoms	0	0	0
	None	0	0	10 (20)

Numbers of cases and specimens (in brackets) per group are given. For cases of unilateral EH, the unaffected contralateral specimens (no history of otological disease, no EH) are listed separately (^a^). In some cases, one specimen was excluded due to artifactual damage to the ES (^b^), three additional specimens from two cases without information on otological symptoms

### Human temporal bone histopathology

The human TB collection at the Massachusetts Eye and Ear Infirmary contains approximately 2300 histologically processed autopsy specimens as well as the corresponding clinical records. The standardized methods for the histological processing of human TB specimens are described elsewhere [34]. The computer database of clinical records was searched for the key word “endolymphatic hydrops”. A total of 224 specimens were identified. Specimens (114, 50.9%) were excluded from the study for reasons listed in Supplementary Table 3. An investigator blinded to any further histological information classified each case according to the criteria mentioned in the previous paragraph. Control specimens had no history of otological disease (except symmetrical presbyacusis in the corresponding age range), and no obvious histopathological findings in the temporal bone. The severity of EH in each included specimen was rated by an investigator (AHE) according to a previously established four-level (absent, mild, moderate, severe) rating system [46]. The investigator was blind to the ES histopathology and the medical records when assigning the EH ratings. A second investigator (JCA) who was blinded to the severity and group assignment of EH (secondary EH or idiopathic EH), as well as to the medical records, evaluated the integrity of the epithelium in the iES and the eES separately for each case according to a seven-level rating system, which considered the overall epithelial integrity, epithelial cell morphology, and nuclear morphology (Supplementary Table 4).

### Immunohistochemistry

Paraffin sections were deparaffinized in xylenes and hydrated in a descending ethanol series. Celloidin sections were mounted on microscope slides, and the celloidin was removed according to methods described elsewhere [38]. For heat-induced antigen retrieval (HIAR), sections were immersed in 10 mM sodium citrate (pH 6.0), placed in a pressure cooker, and heated in a microwave oven. The celloidin sections were coverslipped before HIAR to avoid section detachment during the heating phase; a detailed protocol for coverslipping mounted tissue sections for HIAR will be provided in a later publication. All sections were then blocked in 5% normal horse serum (NHS) diluted in phosphate-buffered saline (PBS), and subsequently incubated with primary antibodies that were diluted in 1% NHS/PBS. Primary antibodies were visualized using either chromogenic or fluorogenic detection methods. In the former, sections were incubated with biotinylated secondary antibodies for 1 h, followed by application of an avidin-biotin complex (ABC) reagent (Jackson ImmunoResearch, West Grove, PA) for 1 h; an optional amplification step included incubation with biotinylated tyramine for 10 min, followed by incubation with ABC reagent for 30 min [1]. Then, all sections were incubated in diaminobenzidine/hydrogen peroxide in PBS supplemented with 4% 3,3′-diaminobenzidine (DAB; Sigma) for two to ten minutes. Hematoxylin was used to stain the cell nuclei. The slides were then dehydrated in an ascending ethanol series, cleared with xylenes, and mounted for microscopic analysis (detailed protocols for individual experiments are given in Supplementary Table 1). For immunofluorescent labeling of primary antibodies, the sections were (1) incubated with fluorochrome-conjugated secondary antibodies for 1 h and/or (2) incubated with biotinylated secondary antibodies followed by incubation with ABC reagent for 1 h and then with fluorochrome-conjugated streptavidin for 30 min. The sections were coverslipped in Vectashield mounting medium with DAPI (Vector Laboratories, Burlingame, CA). All primary and secondary antibodies were diluted in 1% NHS/PBS. All incubation steps were performed at RT.

### Microscopic analysis

DAB-labeled sections were analyzed using an Olympus BX51 microscope (Olympus, Tokyo, Japan) with an Olympus DP70 digital camera (Olympus). Analysis of fluorescent-labeled sections was performed using a Leica TCS SP5 or a Leica TCS SP8 confocal microscope (Leica, Mannheim, Germany).

### Quantification of DAB immunolabeling

Immunolabeled sections of the murine ES were used for counting the numbers of DAB-positive epithelial cells in the eES portion. For each primary antibody, counts were performed on three immunolabeled sections that were derived from different animals, and the mean numbers and standard deviations of labeled cells along the eES were determined. In immunolabeled sections of the human ES, the intensity and area of DAB labeling in the epithelium of the iES and eES portions was compared. Therefore, in each immunolabeled section, three microscopic images were taken in different regions of the iES and three in the eES. The software ImageJ [44] was used to measure the DAB-labeled epithelial area in each image. The same color intensity threshold was used to analyze images that were taken from the same tissue section. Each type of immunolabeling was performed on three nonconsecutive sections from the same specimen in order to determine the mean values and standard deviations of the DAB-stained epithelial area in the iES and eES portions. The ratio of mean DAB labeled epithelium in the eES (*A*_eES_) and the iES (*A*_iES_) is given for each antibody.

### Proximity ligation assay

Sections were deparaffinized and HIAR was performed using the same protocols that were applied prior to immunohistochemical labeling experiments. Sections were then incubated ON at RT with primary antibodies that were diluted in 1% NHS/PBS. For PLAs, the Duolink Red Fluorescence Kit (Sigma) was used according to the manufacturer’s instructions. Sections were coverslipped with Vectashield mounting medium with DAPI (Vector Laboratories) and analyzed using a Leica TCS SP5 confocal microscope (Leica). Puncta of PLA signal per epithelial cell in the eES portion were counted using the software “BlobFinder” [2]. For each experimental condition (standard Na^+^, low Na^+^, high Na^+^), at least six sections from three different animals were analyzed.

### Statistics

Statistical evaluation was performed using GraphPad Prism (v 7.0a; GraphPad Software, La Jolla, California, USA) as indicated in the figure legends. A *p* value of less than 0.05 was considered significant. All Student’s t-tests were unpaired and two-sided. ANOVA was one-way and was always used with Tukey’s honest significant difference (HSD) post hoc test.

### Compliance with ethical standards

All animal and human procedures were approved by the Institutional Animal Care and Use Committee (IACUC) and the Human Research Protections Program (HRPP) of the Massachusetts Eye and Ear Infirmary, respectively.

## Results

### ALDO-regulated Na^+^ transport proteins in the murine ES

As schematized in Fig. 1b, the molecular cascade mediating ALDO-responsive control of Na^+^ reabsorption in the kidney’s “aldosterone-sensitive distal nephron” (ASDN) include the mineralocorticoid receptor (MR), which, upon ALDO binding, translocates to the nucleus and upregulates serum/glucocorticoid-regulated kinase 1 (SGK1). SGK1, in turn, increases the expression and activity of the epithelial sodium channel (ENaC), the renal outer medullary potassium channel (ROMK) and the sodium/potassium ATPase (NKA) by inhibiting WNK lysine-deficient protein kinase 4 (WNK4) and E3 ubiquitin ligase NEDD4-2 (NEDD4-2), which, in the absence of ALDO, promote proteolytic degradation of ENaC, ROMK and NKA. The membrane-bound enzyme transmembrane protease serine 3 (TMPRSS3) is an ALDO-independent positive regulator of ENaC. 11β-Hydroxysteroid dehydrogenase isoenzyme 2 (11β-HSD2), the signature enzyme of mineralocorticoid target tissues, prevents overstimulation of MR by constitutively converting the biologically active compound cortisol (*F*) to the inactive compound cortisone (*E*).

Immunolocalization of these molecular players in the ALDO cascade was conducted in the endolymphatic duct and the intraosseous ES (iES) and extraosseous ES (eES) of the murine inner ear (Fig. 1a). No immunoreactivity for these proteins was detected in the endolymphatic duct (data not shown). In the iES, only weak and sporadic labeling was observed (Fig. 1c). In the operculum (arrows in Fig. 1c), at the transition from the iES to the eES, the number of labeled epithelial cells abruptly increased, continuing to increase towards the distal eES (Fig. 1d). Quantification of immunolabeled cells confirmed the increased expression of all ALDO-regulated Na^+^ transport proteins along the proximal-to-distal axis of the eES (Fig. 1e). Confocal microscopy analysis demonstrated subcellular localization in the membranous, cytoplasmic, and nuclear domains, consistent with previous reports in other tissues. Figure 1f–f″ shows MR labeling in the nucleus, γENaC labeling in the apical membranes and NKA labeling in the basolateral membranes of the eES, a pattern consistent with transcellular Na^+^ transport across the eES epithelium. The presence of ALDO-regulated Na^+^ transport proteins, in particular ENaC and NKA, in murine and human ES epithelial cells is in agreement with previous reports (reviewed in [25, 35]). Labeling results for other ALDO-regulated proteins in the murine eES and in the murine kidney tissue (positive controls) are shown in Supplementary Figs. 1 and 2, respectively.

### Na^+^ intake and regulation of Na^+^ transport proteins in the murine eES

Na^+^ transport in ALDO-sensitive epithelia, such as the renal distal nephron, is controlled via MR and its intracellular downstream effectors and inhibitors. Those downstream molecules include SGK1 (effector), as well as NEDD4-2 (inhibitor) and Wnk4 (activator or inhibitor), which regulate the expression, membrane localization, and activity of the ion transport proteins. With elevated plasma Na^+^, a decreased amount of ALDO is released to bind to MR in epithelial cells, and constitutively active NEDD4-2 interacts with ENaC and ROMK channel units, leading to their ubiquitination and degradation from the apical cell membrane. Transepithelial reabsorption of Na^+^ is thereby decreased and excess Na^+^ excreted. In the event of lowered plasma Na^+^, increased MR binding by ALDO leads to activation of SGK1, which directly inhibits NEDD4-2 and thereby prevents degradation of membranous ion transporters. Transepithelial Na^+^ reabsorption from the urine is then increased.

**Fig. 2:**
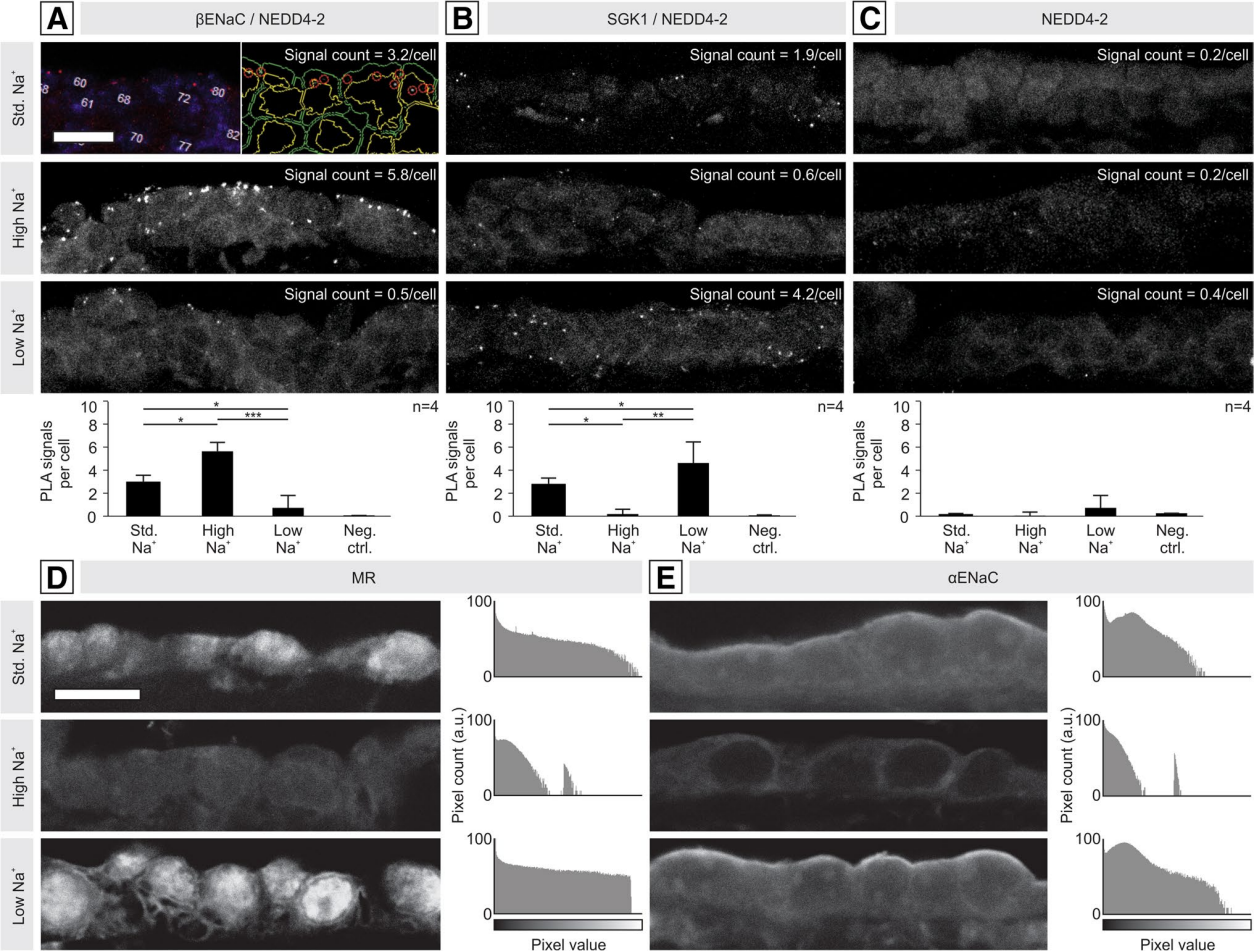
Na^+^ transport proteins in the murine eES after 7 days on a standard-Na^+^ (0.14%), high-Na^+^ (4.0%), or low-Na^+^ diet (0.04%). **a**–**c** Analysis of protein–protein interactions by proximity ligation assays (PLA) in the eES for **a** E3 ubiquitin ligase NEDD4-2, which ubiquitinylates ENaC subunits and facilitates degradation in the absence of ALDO stimulation (high-Na^+^); **b** serum/glucocorticoid-regulated kinase 1 (SGK1), which, upon ALDO stimulation (low Na^+^), inhibits NEDD4-2 and increases expression of ENaC-subunits; and **c** negative controls using a single antibody (anti-NEDD4-2), which produced signal counts close to zero. Automated PLA-signal quantification was performed with BlobFinder software [2] as shown in **a**. In each image (*n* = 4 per group), nuclei were counted in the DAPI channel (left), and the number of PLA signal puncta (red dots in left panel, red circles in right panel) were counted; the means and standard errors are shown in the histograms below each image. Yellow and green outlines represent nuclear and cell borders, respectively, as generated by BlobFinder. **d**, **e** Immunolabeling of the mineralocorticoid receptor (MR) and αENaC in the distal eES. Under low-Na^+^ conditions, nuclear MR labeling was increased, indicating increased nuclear translocation of MR receptors, and αENaC labeling was increased both in strength and in polarization to the apical membrane. Histograms for each image are given. Statistics: one-way ANOVA; *, *p* ≤ 0.05; **, *p* ≤ 0.01; ***, *p* ≤ 0.001. Scale bars: **a**–**e** 20 µm

To investigate regulation of Na^+^ transport in the murine eES, we fed mice on diets with different Na^+^ content for 7 days. Measures of metabolic balance and ALDO concentration showed that animals on high Na^+^ had significantly increased fluid intake and urine output and significantly decreased plasma/urine ALDO levels compared to animals on a low-Na^+^ diet; mice on a standard-Na^+^ diet showed intermediate values (Supplementary Fig. 3, A–D). Proximity ligation assays (PLAs) were performed on eES tissue sections from the three experimental groups to quantify protein–protein interactions in the MR downstream cascade. For NEDD4-2 and βENaC, protein–protein interactions (PLA-signal counts) were increased on the high-Na^+^ diet compared to the other two conditions (Fig. 2a), indicating increased NEDD4-2-mediated ubiquitination of βENaC in the eES under high Na^+^ (low plasma ALDO). This change is expected to reduce the membrane abundance of ENaC channels and other ion transport proteins, such as ROMK and NKA. For SGK1 and NEDD4-2, PLA-signal counts were significantly increased under low-Na^+^ conditions (Fig. 2b), indicating increased SGK1-mediated inhibition of NEDD4-2 function. This change is expected to increase the membrane abundance of ion transport proteins due to decreased NEDD4-2-mediated ubiquitination. Negative controls (incubation with NEDD4-2 antibody only) showed PLA-signal counts close to zero under all dietary conditions (Fig. 2c). These results were similar to those reported for the renal distal nephron under different Na^+^ loads [29].

Next, we used immunostaining to assess the protein expression levels and subcellular localization of ion transport proteins in the murine eES under different Na^+^ loads. For MR, we detected increased nuclear labeling in the eES in the presence of low Na^+^ and reduced nuclear labeling in the presence of high Na^+^ (Fig. 2d). Since the anti-MR antibody recognizes ALDO-bound protein, label intensity is a semiquantitative measure of binding between MR and ALDO. For αENaC, we saw increased and apically polarized labeling in the eES under low Na^+^ and weaker (almost absent) labeling under high Na^+^ (Fig. 2e). The observed changes were in line with those reported for the kidney [29]. Qualitative immunolabeling patterns for other ALDO-regulated proteins (βENaC, γENaC, ROMK, NKA), as well as corresponding data from murine kidney sections (positive controls) from all three experimental conditions, are shown in Supplementary Fig. 3, E–J.

### ALDO-regulated Na^+^ transport proteins in the human eES

**Fig. 3:**
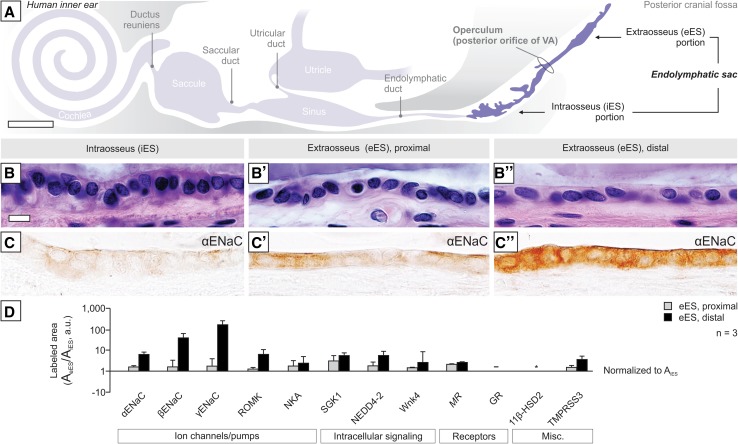
Immunolocalization of ALDO-regulated proteins in the normal human ES. **a** Schematic of the endolymphatic compartments, including the iES and eES, in human (adapted from [34]). **b**–**b″** Morphology of the iES (**b**), and the proximal (**b**′) and distal eES (**b**″). **c**–**c″** Immunostaining for αENaC shows a proximal-to-distal gradient in the iES (**c**), proximal eES (**c**′) and distal eES (**c**″). **d** Semiquantitative analysis of labeling intensity for ALDO-regulated proteins in the proximal and distal eES. Labeled epithelial area (means ± SEM; *n* = 3) was normalized to the iES. GR labeling was not detected in the ES. Scale bars: **a** approximately 2 mm; **b**–**c**″ 20 µm

As in mouse, the human ES is divided into an iES within the vestibular aqueduct and an eES in the posterior cranial fossa (Fig. 3a). The epithelium in the different portions of the human ES exhibits distinct morphologies, i.e., tubular columnar cells in the iES (Fig. 3b), transitional columnar-to-cuboidal cells in the proximal eES (near the operculum; Fig. 3b′), and uniformly cuboidal cells in the distal eES (Fig. 3b″). As in the murine ES, in human, immunoreactivity for ALDO-regulated proteins was detected only in the proximal and distal eES. All proteins exhibited proximal-to-distal gradients within the eES, as demonstrated qualitatively for αENaC (Fig. 3c–c″) and semiquantitatively in Fig. 3d for all ALDO-regulated proteins. Subcellular localization patterns for all ALDO-regulated proteins in the distal eES, as well as immunolabeling results from the human renal tubular epithelium (positive controls), are shown in Supplementary Fig. 4.

### Degeneration of the human eES in idiopathic EH

**Fig. 4:**
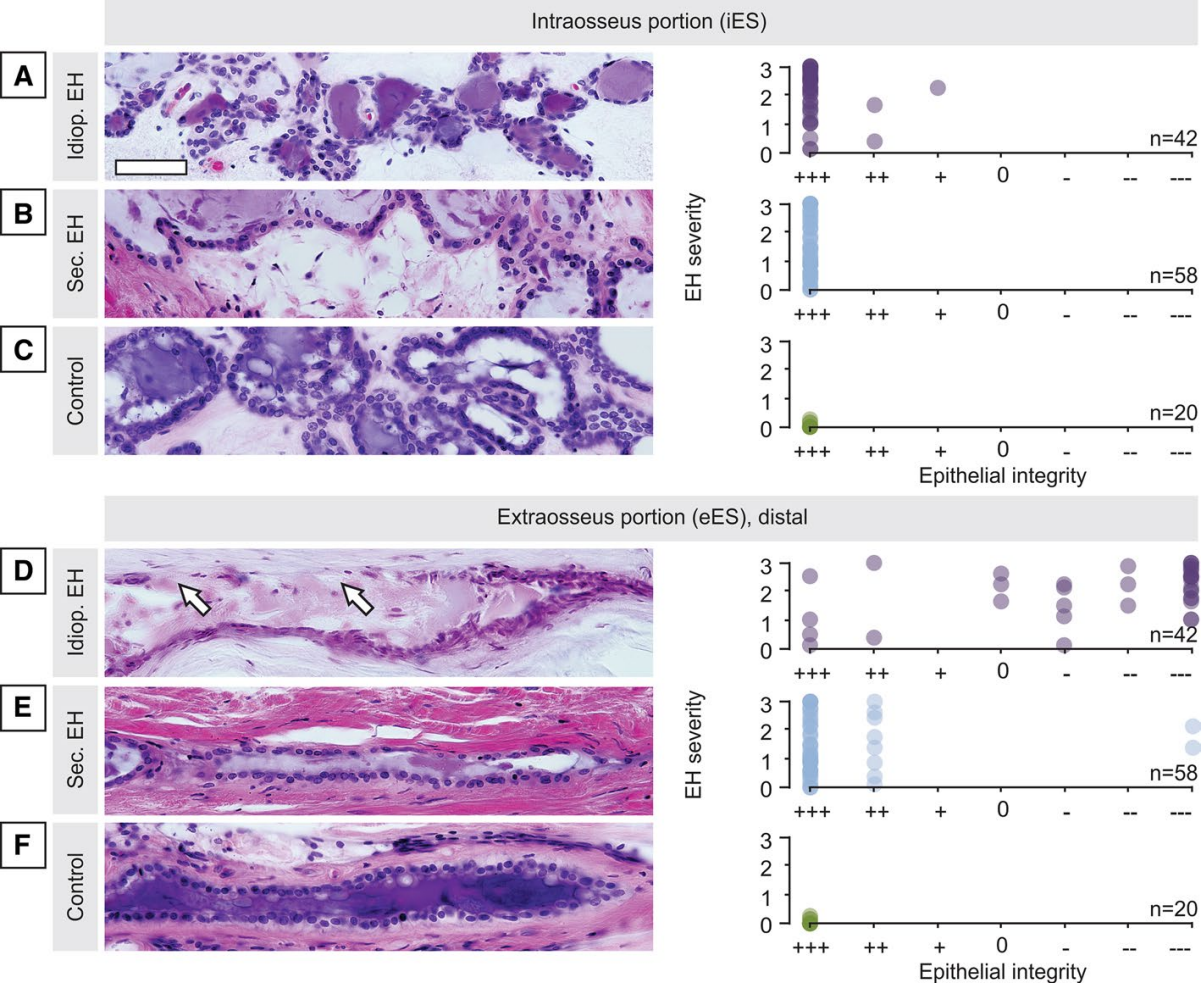
Degenerative pathology of the eES in idiopathic EH. Epithelial integrity in the iES (**a**–**c**) and eES (**d**–**f**) in idiopathic EH (*n* = 42), secondary EH (*n* = 58), or in controls (*n* = 20) as indicated on the extreme left. Representative images of the iES (**a–c**) and eES (**d–f**) for each group; note the degenerative epithelial pathology in idiopathic EH (arrows in **d**). Ratings of epithelial integrity (+++ intact to − − − severely damaged/absent) are plotted against EH severity for all cases. Scale bars: **a–f** 50 µm

From the human pathology collection at the Massachusetts Eye and Ear Infirmary, we identified three groups: 42 specimens from patients with idiopathic EH, 58 with secondary EH, and 20 controls without a history of otological disease. The classification criteria are described in Materials and Methods. In a blinded fashion, epithelial integrity in iES and eES was evaluated in the archival hematoxylin and eosin (H&E)-stained sections on a 7-point scale, from completely intact (+++) to completely absent (− − −). The iES was largely intact (+, ++, +++) in all three groups. Examples for each group and corresponding ratings are shown in Fig. 4a–c. In the eES epithelium, mild to severe epithelial damage (0, −, − −, − − −) was seen in most (72.7%) specimens from patients with idiopathic EH (Fig. 4d). eES pathology mainly comprised shrunken or expelled epithelial cells, pyknotic nuclei, and fibrosis in areas of complete epithelial loss. By contrast, in cases of secondary EH (Fig. 4e) and in control cases (Fig. 4f) the eES epithelium was largely intact (more examples from all three groups in Supplementary Figure 5). In summary, among patients with idiopathic EH, 95.8% (23/24) had a diagnosis of MD, and 54.2% (13/24) exhibited eES degeneration on the affected side(s). Conversely, only 7.7% (3/39) of patients with secondary EH had MD-like symptoms, and only 2.6% (1/39) of these cases showed degeneration in the eES (one patient, both sides affected). None of the controls had a history of otological symptoms or exhibited degeneration of the ES epithelium (see also Table 1).

Degeneration of the eES was also found in a rare adolescent case in the collection. This 13-year-old had symptoms consistent with unilateral definite MD in the early “fluctuating” stage, including hour-long vertigo spells and right-sided fluctuating hearing loss. Consistent with the clinical diagnosis, histopathology of the right inner ear showed severe EH in all endolymphatic compartments. The eES portion exhibited severe degenerative changes (Supplementary Fig. 6). No signs of age-related degenerative change or typical changes associated with late-stage MD were noted in the inner ear.

### Hypoplasia of the human ES in idiopathic EH

**Fig. 5:**
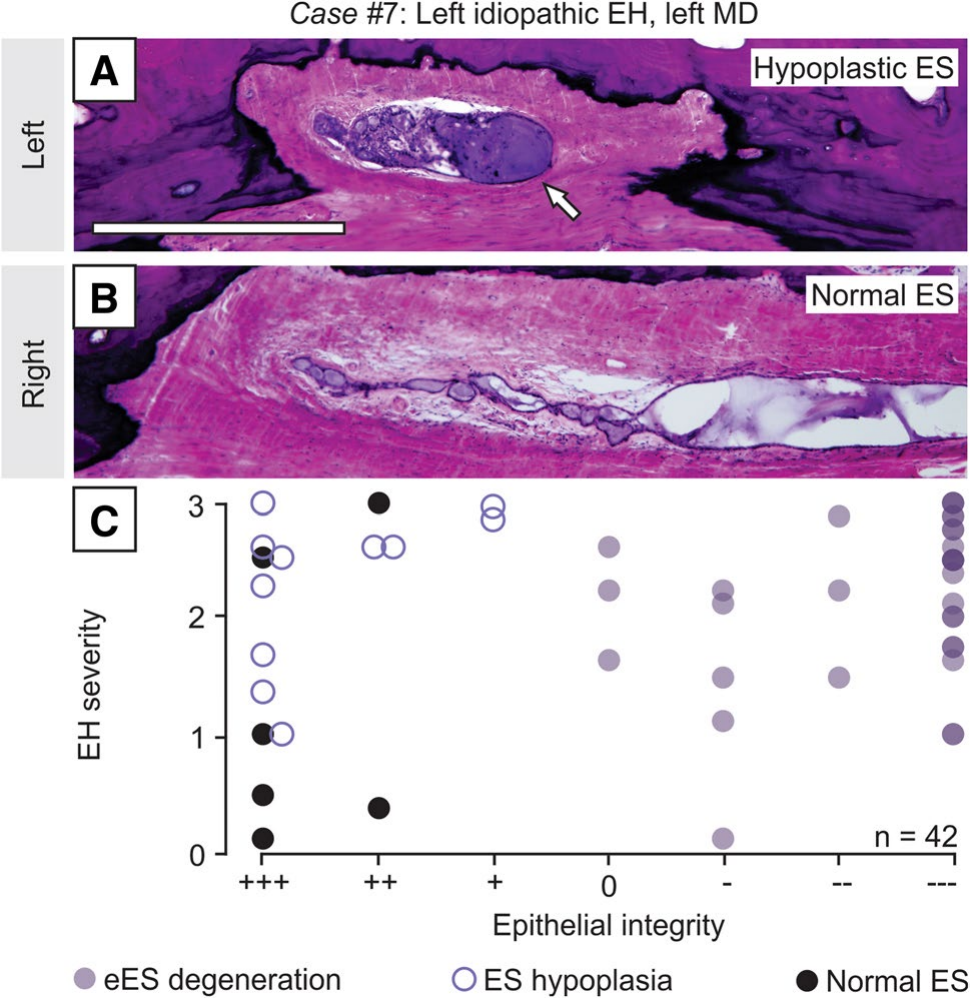
ES hypoplasia in idiopathic EH cases without degenerative ES pathology. **a**, **b** ES morphology in the right (**a**) and left inner ear (**b**) in a patient with left severe idiopathic EH and a diagnosis of left definite MD; note the hypoplastic left ES, with the distal end of the ES in the operculum (arrow in **b**). **c** Among the 14 specimens with idiopathic EH that did not show eES degeneration (data points in box), 11 specimens (78.6%) demonstrated ES hypoplasia (open circles). Same data as presented in Fig. 4a. Scale bars: **a**, **b** 50 µm

Among the specimens from cases of idiopathic EH, 11 were lacking a discernable eES portion and were, therefore, not included in the analysis in Fig. 4. Closer examination revealed a hypoplastic ES morphology in those 11 specimens. As shown in Fig. 5a and b, a patient with severe left-sided idiopathic EH and a clinical diagnosis of left MD, the left ES terminated prematurely in the operculum, and the entire eES portion was missing (Fig. 5a; arrow marks the distal end of the ES in the operculum). In the unaffected right inner ear, a normal eES portion with intact epithelial cells was noted (Fig. 5b). Evaluation of epithelial integrity showed an abnormally pleomorphic, predominantly squamous epithelium, but no signs of epithelial degeneration in any hypoplastic ES (Fig. 5c, open circles indicate specimens with ES hypoplasia; other data points as shown in Fig. 4d). ES hypoplasia was present as unilateral (e.g., case in Fig. 5a, b, with left ES hypoplasia) or bilateral pathology and was always associated with an ipsilateral MD diagnosis and ipsilateral EH. ES hypoplasia was never seen in specimens from patients with secondary EH or from controls. Thus, in summary, either degeneration of the eES (13/24 cases) or ES hypoplasia (9/24 cases) was found in 95.8% of cases with idiopathic EH (with or without clinical MD) but in virtually no case with secondary EH or no EH (only 1 case with secondary EH showed ES degeneration; see also Table 1).

### Loss or absence of ALDO-regulated proteins in degenerative and hypoplastic ES pathology

**Fig. 6:**
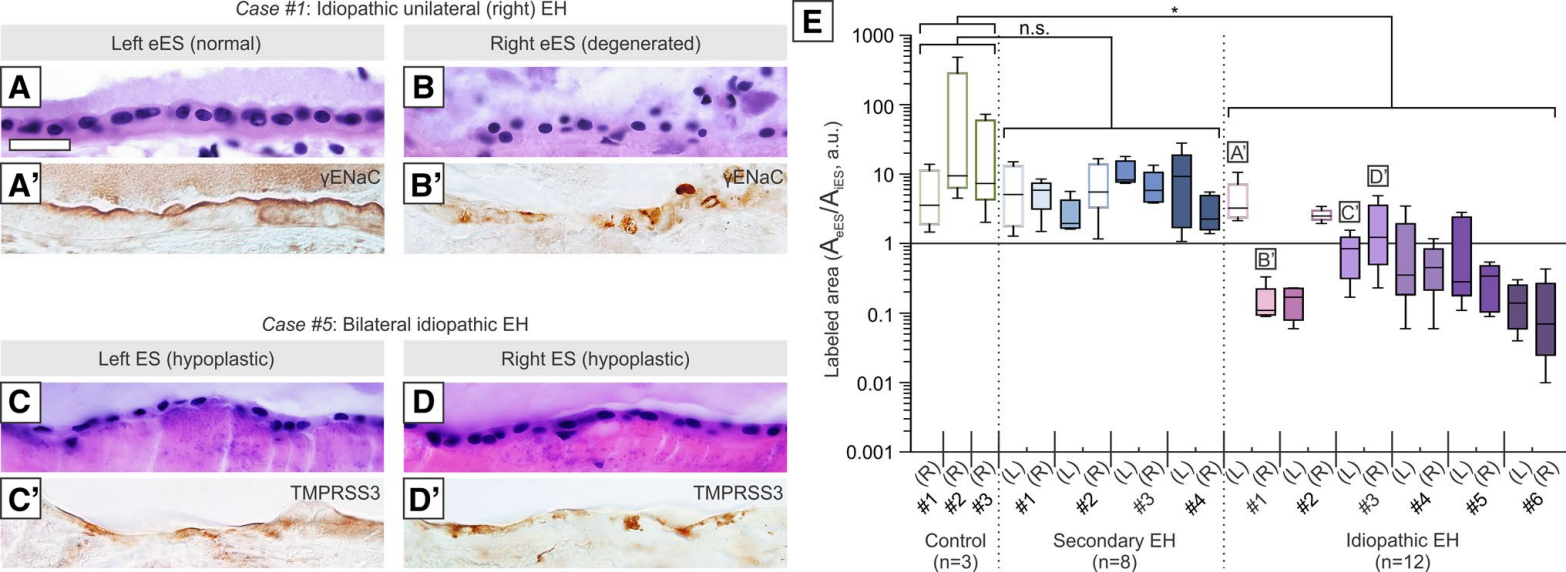
Loss or absence of ALDO-regulated proteins in degenerative and hypoplastic ES pathology. **a**–**b′** A case of unilateral (right) idiopathic EH and ipsilateral degenerative ES pathology: on the left ES, morphology (**a**) and γENaC immunostaining (**a**′) are normal in the eES. In contrast, the right ES showed severe epithelial degeneration (**b**) and loss of apically polarized γENaC immunostaining (**b**′). **c**–**d′** A case of bilateral idiopathic EH and bilateral hypoplastic ES pathology: both ESs exhibited an abnormal squamous epithelium (**c**, **d**) with weak immunostaining for ALDO-regulated proteins. Immunostaining for TMPRSS3 is shown (**c**′, d′). **e** Ratios of labeled epithelial area in eES (normalized to the iES) as determined for ENaC (α, β, γ), ROMK, and TMPRSS3 in cases of idiopathic EH (n = 6), secondary EH (n = 4) and controls (n = 3), as indicated along the *x*-axis. All ratios determined for one specimen are summarized in a box plot. Hollow box plots indicate specimens without EH, including controls and the unaffected sides from EH cases. Statistics: **e** one-way ANOVA; *, *p* ≤ 0.05; *ns* not significant. Scale bars: **a**–**d**′ 50 µm

To investigate expression patterns of ALDO-regulated proteins in ESs affected by degenerative or hypoplastic pathology, we immunostained ES epithelia from selected human cases. A patient with right idiopathic EH and degenerative pathology of the right eES had a clinical history of bilateral sensorineural hearing loss (worse on the right), rare episodes of tinnitus, and intermittent “balance problems”, although the record did not mention MD (Fig. 6a, b′). In the left ear, the eES showed an intact epithelium (Fig. 6a), normal apically polarized immunolabeling for γENaC (and other ALDO-regulated proteins, data not shown), and normal proximal-to-distal immunolabeling gradients (Fig. 6e, box plot labeled a′). By contrast, in the right ear, the eES epithelium showed severe degenerative changes (Fig. 6b); weak, nonpolarized immunolabeling (Fig. 6b′); and loss of the proximal-to-distal immunolabeling gradients (Fig. 6e, box plot labeled B′). Another patient with bilateral idiopathic EH and bilateral ES hypoplasia had a clinical history of bilateral MD (Fig. 6c, d′). In both ears, the most distal ES (in the operculum) showed a pleomorphic, squamous epithelium (Fig. 6c, d); weak, nonpolarized immunolabeling for TMPRSS3 (Fig. 6c′, d′) and ALDO-regulated proteins (data not shown); and loss of the proximal-to-distal immunolabeling gradients (Fig. 6e, box plots labeled c′ and d′). Data for three more cases of idiopathic EH and secondary EH are shown in Supplementary Fig. 7. Immunohistochemical analysis of ALDO-regulated proteins in three controls, four patients with secondary EH, and six patients with idiopathic EH (including Cases #1 and #5; Fig. 6e) showed normal proximal-to-distal immunolabeling gradients (*A*_eES_/*A*_iES_ ratios > 1) in all controls and all patients with secondary EH. In contrast, all specimens from patients with idiopathic EH exhibited *A*_eES_/*A*_iES_ ratios ≤ 1, indicating loss of the normal proximal-to-distal gradients. Notably, in all unilateral idiopathic EH cases, the unaffected contralateral side (unshaded box-and-whisker plots in Fig. 6e) exhibited an intact eES epithelium, normal proximal-to-distal label gradients and significant differences between the affected and unaffected sides.

### Clinicopathological correlations suggest prognostically relevant subtypes of MD

**Fig. 7:**
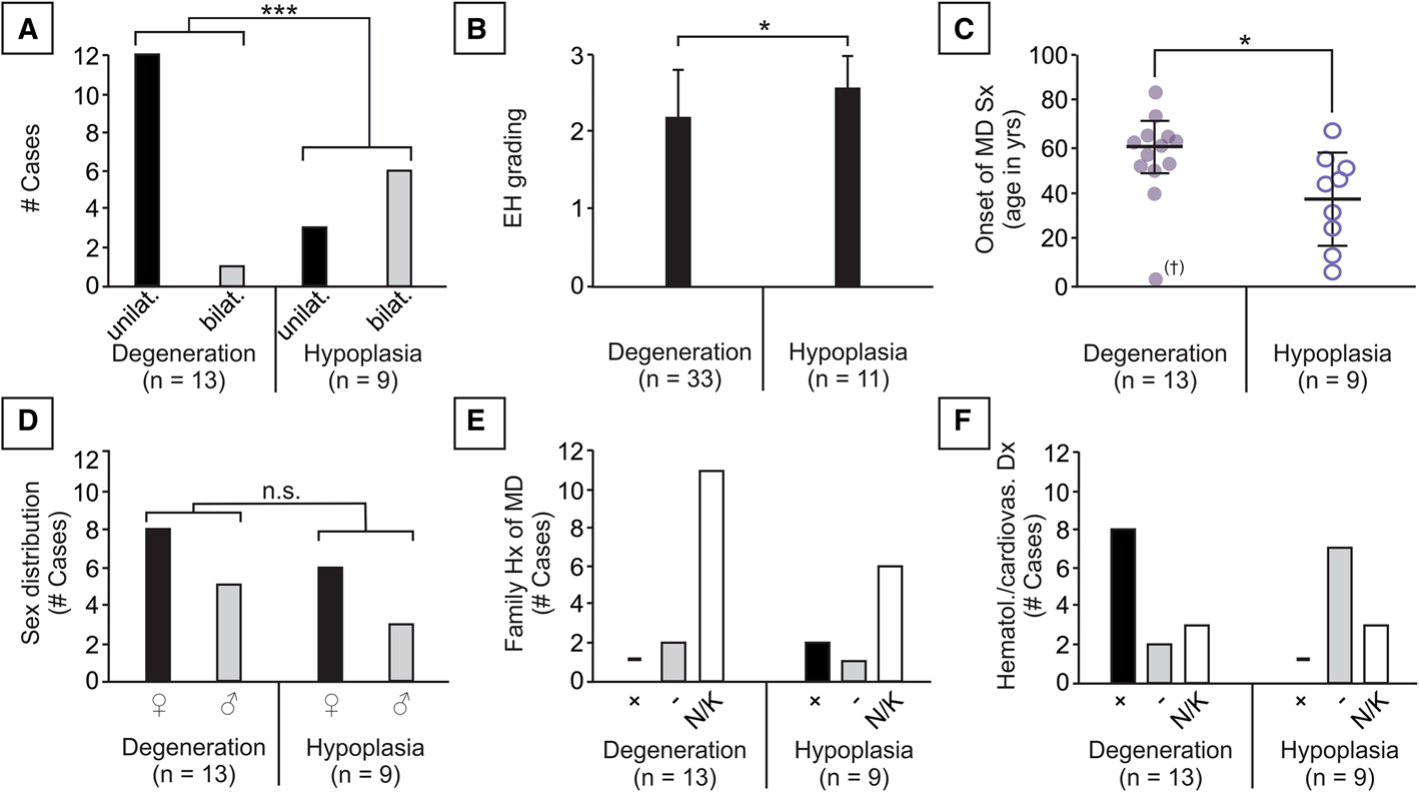
Clinicopathological correlations in patients with idiopathic EH and associated clinical MD. Statistics: **a**, **b**, **d** Fisher’s exact test; **c** two-tailed Student’s *t* test; *ns* not significant; *, *p* ≤ 0.05; **, *p* ≤ 0.01; ***, *p* ≤ 0.001

We show here that idiopathic EH and associated clinical MD can be subdivided according to ES pathology: degeneration or hypoplasia. Both pathologies were linked with distinct clinical traits, according to our retrospective chart reviews. Degenerative ES pathology was associated with unilateral disease (12 out of 13 cases) significantly (*p* = 0.0066) more often than bilateral disease, whereas hypoplasia was found in 6 out of 9 bilateral cases (Fig. 7a). EH was significantly (*p* = 0.021) increased in severity when hypoplastic ES pathology was present (Fig. 7b). Patients with degenerative ES pathology showed significantly (*p* = 0.038) higher age at onset (58.1 ± 20.5 years) than patients with hypoplastic ES pathology (37.7 ± 19.0 years; Fig. 7c). No significant difference was found in the sex ratio between degenerative (1.6 females/male) and hypoplastic pathology (2 females/male; *p* = 1.0; Fig. 7f). A positive family history of MD was reported in only two cases of ES hypoplasia; for most cases, this information was not available in the clinical records (Fig. 7e). Among the reported comorbidities, only patients with degenerative ES pathology had a positive history of hematological or cardiovascular disease diagnosed prior to the first onset of MD symptoms (Fig. 7f; detailed diagnoses are listed in Supplementary Table 5); however, the increased age at MD onset in patients with degenerative ES, as well as incomplete clinical records, may confound this analysis.

## Discussion

**Fig. 8:**
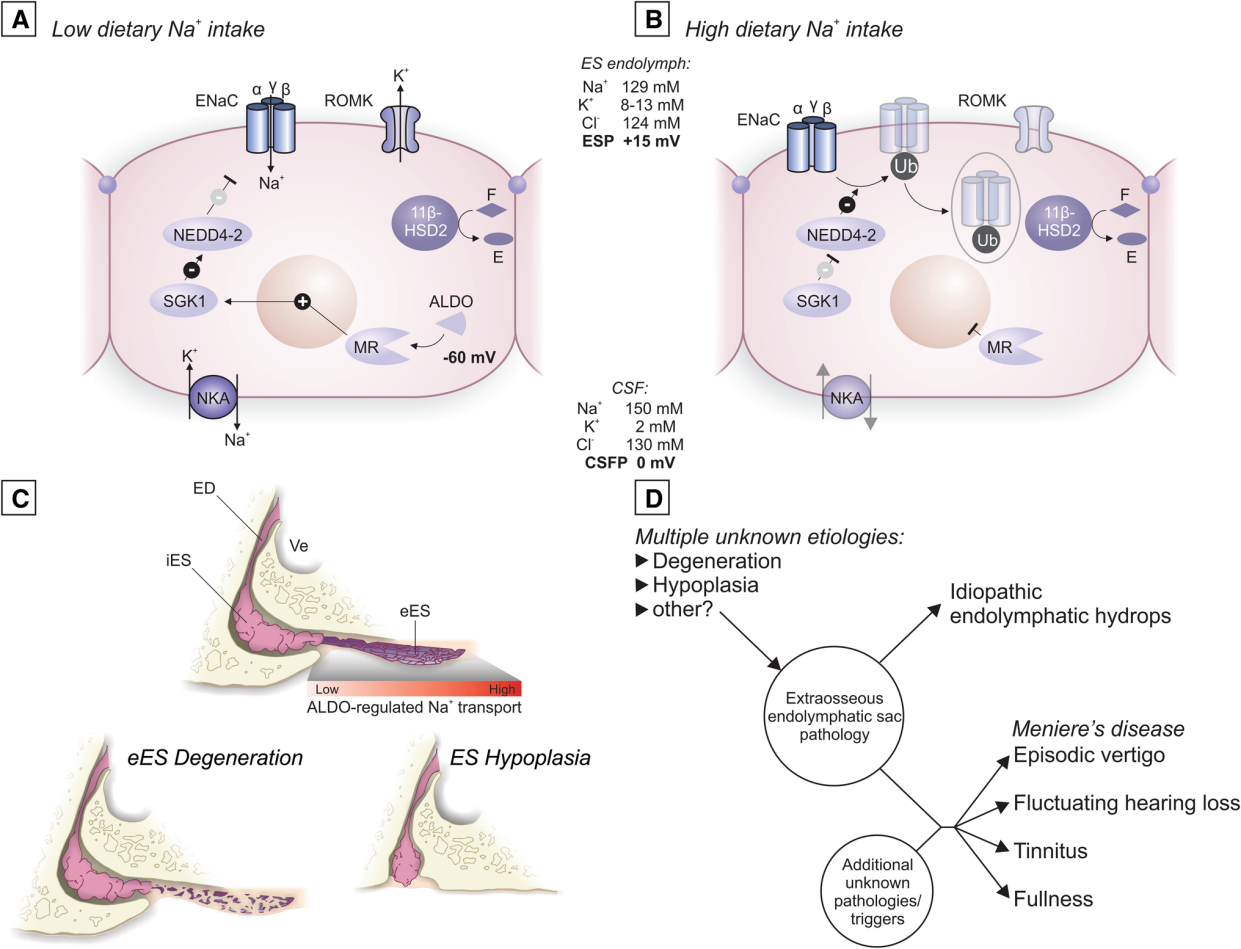
**a**, **b** Schematic of ALDO-regulated ion transport in eES epithelial cells, as identified in the present study. Interaction pathways and presumed effects on transepithelial ion fluxes are depicted under low-Na^+^ (**a**) and high-Na^+^ (**b**) conditions. *ESP* endolymphatic sac lumenal potential, *CSF* cerebrospinal fluid, *CSFP* CSF potential. **c** Schematic of normal human ES morphology (upper panel), and the two pathologies of the eES seen in idiopathic EH with clinical MD, i.e., epithelial degeneration (left panel) or hypoplasia (right panel). *Ve* vestibule, *ED* endolymphatic duct. **d** Schematic of the hypothesis for idiopathic MD (adapted from [33, 24]), modified based on findings from the present study

Previous histopathological studies on the underlying pathology of idiopathic EH and MD found widespread mild-to-moderate degenerative changes in the inner ear (reviewed in [34]), but those changes were all ultimately deemed to be either secondary to a long-standing disease process or not disease-specific. In summary, a specific inner ear pathology that provides a conclusive link to the etiology of idiopathic EH and associated clinical MD symptoms has not yet been identified. Here, we show for the first time that two pathologies affecting the eES epithelium are consistently associated with idiopathic EH and MD: degeneration and hypoplasia. We further demonstrate that the normal eES epithelium is sensitive to changes in systemic Na^+^ intake and harbors key molecular features for aldosterone-regulated transepithelial Na^+^ transport (Fig. 8a, b). We therefore consider loss/absence of the eES and its ion transport function to be critically involved in the etiology of idiopathic EH and MD symptoms.

We demonstrated here that degenerative pathology (Fig. 8c, lower left panel) and developmental hypoplasia of the eES (Fig. 8c, lower right panel) are consistently and specifically associated with idiopathic EH, since both pathologies were found in 13/14 patients (95.8%) with idiopathic EH but only 1/39 patients (2.6%) with secondary EH and no controls. This finding suggests that patients with idiopathic EH and a clinical history of MD are substantially different from those with secondary EH and secondary Meniere’s syndrome with regard to their suspected etiopathology. Our results thereby support the previously established concept of primary and secondary hydropic inner ear diseases [21] and, for the first time, provide a clear distinction between those two disease categories on the pathological level.

To be considered etiological factors for idiopathic EH and clinical MD, both eES pathologies must be present prior to the appearance of EH and clinical symptoms. In the case of degenerative pathology, we identified the case of an adolescent MD patient presenting with early-stage fluctuating symptoms at the time of death (Supplementary Fig. 6). Severe degenerative change in the eES in the clinically affected inner ear was the only pathology clearly recognizable as premortem pathology, indicating that eES degeneration is not a secondary change that occurs in the course of the disease. In the case of ES hypoplasia, the pathology presumably manifests during early (fetal) development and is therefore manifested decades before patients start to present clinical symptoms. A direct etiological link between loss of the eES and EH is supported by animal studies, in which surgical separation [26], or destruction of the eES [12] resulted in the development of EH. Notably, these animals, despite developing EH, do not present Meniere’s-like fluctuating vestibular symptoms [26, 12]. We, therefore, consider eES pathology in humans to be a necessary but not a sufficient etiological factor in the pathogenesis of clinical MD (Fig. 8c).

We demonstrated here that the eES epithelium shares key molecular features for ALDO-regulated transepithelial Na^+^ transport with other fluid-transporting epithelia, such as the ALDO-sensitive distal nephron (ASDN) in the kidney (Fig. 1b and Fig. 8a, b). Since Na^+^ is the major extracellular cation and the prime determinant of extracellular fluid volume in the body, the ASDN plays a crucial role in controlling the whole-body hydration state by matching the total urinary Na^+^ excretion to the dietary Na^+^ intake [3]. Despite this homeostatic function of the renal ASDN, sudden fluctuations in blood Na^+^ levels occur, e.g., after ingestion of a high-salt meal, and are instantaneously transmitted to the inner ear fluid compartments due to the high ionic permeability of the blood-labyrinth barrier [28, 49]. We propose that the eES epithelium actively and dynamically resorbs Na^+^ from the endolymph in the ES lumen, which, compared to the endolymph in the cochlea and the vestibule, has a high Na^+^ concentration (101 mM, [9]). This mechanism presumably eliminates excess Na^+^ that passively enters the endolymph upon systemic Na^+^ loading. Thereby, the eES, acting synergistically to the renal ASDN as the primary mediator of extracellular Na^+^ and fluid homeostasis, presumably fine-tunes endolymphatic Na^+^ and volume homeostasis by using similar molecular mechanisms to those used by the ASDN.

Loss of eES function presumably impairs the inner ear’s overall (Na^+^) homeostatic capacity, which leads to osmotic changes and ultimately to the generation of EH. In support of this hypothesis, several clinical observations do, in fact, suggest a problem of Na^+^ homeostatic mechanisms in the inner ear from MD patients, i.e., (1) many reported triggers of MD symptoms directly affect extracellular Na^+^ balance, such as sleep deprivation and stress (activation of the renin–angiotensin–aldosterone system), hormonal changes, and dietary indiscretions with respect to water and Na^+^ intake; (2) a commonly applied first-line treatment that successfully alleviates or ameliorates acute episodic symptoms in many MD patients is a Na^+^-restricted diet, in which Na^+^ is evenly spread across meals to avoid a large bolus at any time [45, 10, 18]; and, moreover, (3) recent long-term Na^+^ balance studies in humans identified Na^+^ storage sites within the human body (skin, muscles, brain), which release Na^+^ independently of daily salt intake, thereby causing infradian fluctuations in extracellular Na^+^ that are not alleviated by renal Na^+^ elimination but require extrarenal—organ-specific—Na^+^ regulatory mechanisms in tissue/organ systems that depend on steady extracellular Na^+^ and volume levels (reviewed in [51]). The eES epithelium presumably provides such a local Na^+^ regulatory mechanism for the inner ear, and its loss may render the auditory and vestibular sense organs vulnerable to internal and external triggers that repeatedly strain and exhaust the inner ear’s impaired homeostatic capacity and thereby elicit the recurrent, episodic symptoms of MD.

The heterogeneous nature of MD with regard to its clinical presentation in individual patients causes several problems, i.e., it is often difficult to reliably diagnose MD, the treatment efficacy in individual patients is unpredictable, and the individual course of the disease cannot be prognosticated. In the attempt to distinguish subgroups of MD patients sharing prognostically relevant clinical features, previous studies used phenotypical features (disease laterality, [6, 8, 14, 15]) and genetic analysis (reviewed in [16]). For the first time, we performed clinicohistopathological correlations in MD that suggest differences in “clinical phenotype” between MD patients with degenerative eES pathology and those with hypoplastic eES pathology. Significant differences were found in the average age at disease onset (later in cases with degenerative pathology), disease laterality (degenerative pathology typically occurs in unilateral disease, while hypoplastic pathology typically occurs in bilateral disease), and EH severity (the severity of EH is, on average, slightly increased in patients with hypoplastic pathology). Another notable finding was that the only cases with a positive family history of MD had hypoplastic pathology, which supports the hypothesis this pathology is potentially of hereditary etiology.

With regard to the therapeutic management of MD, the eES is the target for surgical procedures that were developed with the intention to “drain” the hydropic inner ear and thereby treat the clinical symptoms of MD, either by exposing the eES from the surrounding dural tissue to facilitate fluidic exchange between the ES endolymph and the surrounding CSF space (ES decompression procedure, [40]) or by opening the eES lumen to promote direct outflow of excess endolymphatic fluid from the inner ear (ES shunting procedure, [41, 23]). From the results of the present study, it can be concluded that the abovementioned surgical procedures for MD cannot work as hypothesized, since the eES epithelium in the dura of the posterior cranial fossa is either inaccessible due to ES hypoplasia (present study, [7]) or functionally compromised due to degenerative changes (present study). This finding is in line with the ambiguous clinical outcome that these procedures were shown to have with regard to control of acute MD symptoms [5, 42, 50].

Further studies will address the questions of (1) how the respective ES pathologies can be distinguished in clinical MD patients; (2) whether different ES pathologies are associated with clinically meaningful prognostic subgroups of MD patients; (3) whether diminished eES epithelial function in MD is, in fact, a critical predisposing pathology in the pathogenesis of clinical MD symptoms; and, if so, (4) how eES function can be restored. Moreover, future histopathological studies need to take into consideration newly emerging disease concepts, such as migraine-associated vertigo syndromes, in order to answer the question of whether the clinical (phenotypic) resemblance between those syndromes and “classic” MD is due to similar (etio)pathological traits in the inner ear or whether the eES pathologies described here are a distinguishing feature of MD. However, such studies will require the continued prospective collection of postmortem temporal bone specimens from patients whose clinical history has been carefully documented according to the latest diagnostic consensus criteria.

## Conclusion

This study showed for the first time that idiopathic EH and MD are associated with either of two etiologically different pathologies that cause developmental hypoplasia or degenerative epithelial loss of the eES epithelium in the inner ear. We further demonstrated that the normal eES is a salt-intake-sensitive epithelium that, on the cellular and molecular level, shares features with the salt-absorbing, body fluid volume-regulating ASDN epithelium in the kidney. We therefore propose that the eES epithelium is crucial for the maintenance of endolymphatic Na^+^ and volume homeostasis, and we consider absence/loss of the eES epithelium to be the underlying cause of the development of idiopathic EH, as well as a critical predisposing factor for the development of the clinical symptoms of idiopathic MD. Our clinicopathological correlations indicated that different eES pathologies are associated with different “clinical phenotypes” of MD and therefore may be promising surrogate markers to distinguish prognostic subgroups of MD patients with regard to treatment efficacy and the course of the disease.

## Electronic supplementary material

Below is the link to the electronic supplementary material.

**Supplementary Fig.** **1.** Immunolocalization of ALDO-regulated Na^+^ transport proteins in the murine extraosseous endolymphatic sac (eES). Confocal images of double immunofluorescence labeling of the mineralocorticoid receptor (MR) and subunits α, (**A**), β (**B**), and γ (**C**) of the epithelial sodium channel (ENaC), the renal outer medullary potassium channel (ROMK, **D**), the sodium–potassium ATPase (NKA, **E**), serum/glucocorticoid-regulated kinase 1 (SGK1, **F**), and serine/threonine-protein kinase WNK4 (WNK4, **G**). The ALDO-regulated ion transport proteins, as well as the intracellular signaling molecules SGK1 and WNK4, are localized in MR-positive eES epithelial cells. No labeling for any of these proteins was found in MR-negative eES epithelial cells (arrows). Double labeling of the E3 ubiquitin ligase NEDD4-2 and the transmembrane protease, serine 3 (TMPRSS3, **I**) demonstrated cellular colocalization in eES epithelial cells. Negative control experiments in which primary antibodies were omitted did not result in any labeling in the ES epithelium (data not shown). Scale bars: 20 µm (TIFF 16655 kb)

**Supplementary Fig.** **2.** Immunolocalization of ALDO-regulated Na^+^ transport proteins in the murine kidney. Confocal images of double immunofluorescence labeling of the mineralocorticoid receptor (MR) and subunits α, (**A**–**A**″), β (**B**–**B**″), and γ (**C**–**C**″) of the epithelial sodium channel (ENaC), the thiazide-sensitive sodium chloride cotransporter (NCC, **D–D**″), the renal outer medullary potassium channel (ROMK, **E**–**E**″), the sodium potassium ATPase (Na,K-ATPase, **F**–**F**″), serum/glucocorticoid-regulated kinase 1 (SGK1, **G**–**G**″), serine/threonine-protein kinase WNK4 (WNK4, **H**–**H**″), and the glucocorticoid receptor (GR, **I**–**I**″). Double labeling of the E3 ubiquitin ligase NEDD4-2 and the transmembrane protease, serine 3 (TMPRSS3, **J**–**J**″). Consistent with previous reports, all investigated proteins were localized in MR-labeled cells of the aldosterone-sensitive distal nephron (ASDN) tubular epithelium. Scale bars: (A–J), 10 µm; (A′–J′), 50 µm; (A″–J″), 50 µm (TIFF 22979 kb)

**Supplementary Fig.** **3.** (**A**–**D**) Results of metabolic balance studies on mice that were kept for 7 days on either a standard-Na^+^ diet (0.4%, Std. Na^+^), a high-Na^+^ diet (4.0%, High Na^+^), or a low-Na^+^ diet (0.04%, Low Na^+^). Diagrams showing the mean daily fluid intake (**A**) and output (**B**) per animal, as well as the mean plasma aldosterone (**C**) and urine aldosterone (**D**) levels per animal, for each of the three experimental groups. (**E**) Representative examples of immunolocalization patterns for βENaC, γENaC, ROMK, and NKA in the distal eES epithelium from mice fed different levels of dietary Na^+^ (continued from Fig. 3 (**D** and **E**)). (**F**–**J**) Representative examples of the immunolocalization patterns of MR (**F**); ENaC subunits α (**G**), β (**H**), γ (**I**); and NKA (**J**) in the kidneys of mice fed different levels of Na^+^. Statistics: (A–D) *, *p* ≤ 0.05; **, *p* ≤ 0.01; ***, *p* ≤ 0.001. Scale bars: (E–J), 20 µm. (TIFF 5899 kb)

**Supplementary Fig.** **4.** Subcellular immunolocalization of ALDO-regulated Na^+^ transport proteins in the human distal eES and in the human kidney. (**A**) In the human eES, strong immunolabeling of ENaC (α, β and γ subunits) was found in the apical membranes (arrowheads) and the subapical cytoplasmic region. SGK1, NEDD4-2, WNK4 and TMPRSS3 demonstrated cytoplasmic labeling patterns (apically polarized for SGK1 and TMPRSS3). NKA labeling was seen predominantly in the lateral and basal membranes. MR and 11β-HSD2 showed a nuclear labeling pattern. No immunoreactivity for GR was found in the human ES. (**B**–**E**) In the normal human kidney (positive control tissue), immunolabeling of ENaC subunits α (B), β (C), and γ (D) was localized in apical membranes of the tubular epithelium in the cortical collecting duct (CCD). NKA was localized in basolateral epithelial membranes in the CCD (E). Immunolabeling of other proteins involved in ALDO-regulated ion transport (ROMK, NCC, MR, GR, SGK1, NEDD4-2) produced very weak labeling in the tubular epithelium (data not shown), which was presumably due to the long postmortem time of the kidney tissue sample (> 10 h). Scale bars: 20 µm (TIFF 26402 kb)

**Supplementary Fig.** **5.** Additional qualitative examples of the epithelial morphology in the iES (**A**–**C′**) and eES (**D**–**F′**) of controls, patients with secondary EH and patients with idiopathic EH. Note the epithelial damage/loss in cases with idiopathic EH (arrows in (**F**) and (**F′**) indicate remnants of eES epithelium next to areas of complete epithelial loss and fibrous replacement). Scale bars: 50 µm (TIFF 6513 kb)

**Supplementary Fig.** **6.** A 13-year-old patient with right-sided definite MD in the early “fluctuating” disease stage. Episodes of rotational vertigo lasted for minutes, were accompanied by a feeling of pressure in the right ear, and recurred frequently over three to five consecutive days, usually in conjunction with a “flu-like” illness. (**A**) Audiograms at 3.5 months (*) and < 2 months (**) before death indicated severe fluctuating (or progressive) low-frequency hearing loss (TAI, time to autopsy interval). The cytocochleogram (quantification of cellular elements; black areas indicate missing cells along the tonotopic axis) for the right cochlea indicated mild scattered cellular loss, most likely caused by the 4-day postmortem time of the specimen (IHC, inner hair cells; OHC, outer hair cells). (**B**) In all endolymph compartments of the affected ear the epithelial membranes (arrows) were distended, indicating endolymphatic hydrops. (**C**–**D′**) Normal iES morphology on the affected side (overview in **C**, detail in **C′**) and normally weak immunolabel for γENaC (overview, **D**; detail, **D′**). (**E**–**F′**) Severe degeneration in the eES of the affected ear (overview, **E**; detail, **E′**; arrows mark the former location of the eES epithelium) and loss of immunoreactivity for ALDO-regulated proteins (overview, **F**; detail, **F′**; arrows mark the corresponding location as in (E)). (**G**–**H′**) Immune cells in the lumenal and perisaccular tissue of the iES (overview, **G**; detail, **G′**), many of which were positive for macrophage-marker IBA1 (**H**–**H′**). The clinically unaffected left inner ear showed no EH (data not shown). The histomorphology of the left eES could not be analyzed due to artifactual tissue damage caused during removal of the left temporal bone specimen. Scale bars: (B), 100 µm; (C, D, E, F, G, H), 50 µm; (C′, D′, E′, F′, G′, H′), 20 µm (TIFF 13723 kb)

**Supplementary Fig.** **7.** Immunohistochemical labeling of ALDO-regulated proteins in human patients with idiopathic or secondary EH. (**A**–**E**) Case #6, with bilateral idiopathic EH and a clinical history of bilateral MD. The iES epithelium on both sides is intact (**A**, **C**) and exhibits normal (weak cytoplasmic) immunolabeling for αENaC (**A′**, **C′**). In contrast, the eES epithelium on both sides showed signs of severe degeneration (**B**, **D**) and very weak, nonpolarized immunolabeling patterns for αENaC (**B′**, **D′**), resulting in loss of the proximal-to-distal expression gradient (A_eES_/A_iES_ ratios < 1) for ENaC (α, β, γ), ROMK and TMPRSS3 in both ESs (**E**). Data from (E) are also shown in Fig. 6E. (**F**–**J**) Case #2, with left-sided moderate sensorineural hearing loss (SNHL) and tinnitus after a history of labyrinthitis in the left ear. Histological analysis revealed EH, most likely secondary to the labyrinthitis, in the left inner ear. The morphology of the iES (**F**, **H**) and the eES (**G**, **I**) was intact on both sides. A normal proximal-to-distal immunolabeling gradient for all investigated Na^+^ transport proteins was detected in both ESs (iES (**F′**, **H′**), eES (**G′**, **I′**); A_eES_/A_iES_ ratios (**J**)). (**K**–**O**) Case #3, with a clinical diagnosis of Cogan’s syndrome and bilateral secondary EH. Otological symptoms were not Meniere-like and included bilateral SNHL, tinnitus, and balance problems that all developed after labyrinthitis. The morphology of the iES (**K**, **M**) and the eES (**L**, **N**) was intact on both sides. A normal proximal-to-distal immunolabeling gradient for all investigated Na^+^ transport proteins was detected in both ESs (iES (**K′**, **M′**), eES (**L′**, **N′**); A_eES_/A_iES_ ratios (**O**)). Statistics: (E, J, O), one-way ANOVA; n.s., not significant. Scale bars: (A–D′, F–I′, K–N′), 50 µm (TIFF 16163 kb)

Supplementary material 8 (DOCX 129 kb)

Supplementary material 9 (DOCX 51 kb)

Supplementary material 10 (DOCX 56 kb)

Supplementary material 11 (DOCX 226 kb)

Supplementary material 12 (DOCX 48 kb)

## Abbreviations

11β-HSD2: 11-β-Hydroxysteroid dehydrogenase 2
ABC: Avidin–biotin complex
*A*_eES_/*A*_iES_: Ratio of DAB-labeled area in the eES and iES
ALDO: Aldosterone
ASDN: Aldosterone-sensitive distal nephron
BT: Biotinylated tyramine
CSF: Cerebrospinal fluid
ED: Endolymphatic duct
eES: Extraosseous portion of the endolymphatic sac
EH: Endolymphatic hydrops
ENaC: Epithelial sodium channel
ES: Endolymphatic sac
F: 10% Formalin, neutral buffered
FA: 10% Formalin + 1% glacial acetic acid
FG: 10% Formalin + 0.2% glutaraldehyde
FGA: 10% Formalin + 1% glacial acetic acid + 0.2% glutaraldehyde
GR: Glucocorticoid receptor
HIAR: Heat-induced antigen retrieval
IBA1: Ionized calcium-binding adapter molecule 1
iES: Intraosseous portion of the endolymphatic sac
IHC: Inner hair cells
K^+^: Potassium
MD: Meniere’s disease
MR: Mineralocorticoid receptor
Na^+^: Sodium
NCC: Thiazide-sensitive sodium/chloride cotransporter
NEDD4-2: E3 ubiquitin ligase enzyme
NHS: Normal horse serum
NKA: Na^+^/K^+^-ATPase
OHC: Outer hair cells
PLA: Proximity ligation assay
ROMK: Renal outer medullary potassium channel
SGK1: Serum/glucocorticoid-regulated kinase 1
SNHL: Sensorineural hearing loss
SSC: Superior semicircular canal
TAI: Test-autopsy interval
TMPRSS3: Transmembrane protease serine 3
VA: Vestibular aqueduct
WNK4: Serine/threonine-protein kinase
αENaC: Alpha subunit of the epithelial sodium channel
βENaC: Beta subunit of the epithelial sodium channel
γENaC: Gamma subunit of the epithelial sodium channel

## References

[1] Adams JC (1992). Biotin amplification of biotin and horseradish peroxidase signals in histochemical stains. J Histochem Cytochem.

[2] Allalou A, Wählby C (2009). BlobFinder, a tool for fluorescence microscopy image cytometry. Comput Methods Programs Biomed.

[3] Alpern RJ, Caplan MJ, Moe OW (2013). Seldin and Giebisch’s the kidney: physiology and pathophysiology.

[4] American Academy of Otolaryngology-Head and Neck Foundation, Inc (1995). Committee on hearing and equilibrium guidelines for the diagnosis and evaluation of therapy in Meniere’s disease. Otolaryngol Head Neck Surg.

[5] Bretlau P, Thomsen J, Tos M, Johnsen NJ (1989). Placebo effect in surgery for Meniere's disease: nine-year follow-up. Am J Otol.

[6] Chaves AG, Boari L, Munhoz MSL (2007). The outcome of patients with Ménière’s disease. Braz J Otorhinolaryngol.

[7] Chung JW, Fayad J, Linthicum F, Ishiyama A, Merchant AN (2011). Histopathology after endolymphatic sac surgery for Meniere's syndrome. Otol Neurotol.

[8] Clemmens C, Ruckenstein M (2012). Characteristics of patients with unilateral and bilateral Ménière’s disease. Otol Neurotol.

[9] Couloigner V, Teixeira M, Sterkers O, Ferrary E (1999). *In vivo* study of the electrochemical composition of luminal fluid in the guinea pig endolymphatic sac. Acta Otolaryngol.

[10] Dederding D (1929). Clinical and experimental examination in patients suffering from morbus Meniere including study of problems of bone conduction. Acta Otolaryngol.

[11] Doi M (2010). Salt-sensitive hypertension in circadian clock–deficient Cry-null mice involves dysregulated adrenal Hsd3b6. Nat Med.

[12] Dunnebier EA, Segenhout JM, Wit HP, Albers FWJ (1996). Endolymphatic hydrops after total dissection or cauterization of the distal portion of the endolymphatic sac. ORL.

[13] Ferster APOC, Cureoglu S, Keskin N, Paparella MM, Isildak H (2017). Secondary endolymphatic hydrops. Otol Neurotol.

[14] Frejo L (2016). Clinical subgroups in bilateral Meniere disease. Front Neurol.

[15] Frejo L (2017). Extended phenotype and clinical subgroups in unilateral Meniere disease: a cross-sectional study with cluster analysis. Clin Otolaryngol.

[16] Frejo L, Giegling I, Teggi R, Lopez-Escamez JA, Rujescu D (2016). Genetics of vestibular disorders: pathophysiological insights. J Neurol.

[17] Friberg U, Stahle J, Svedberg A (1984). The natural course of Meniere’s disease. Acta Otolaryngol.

[18] Furstenberg AC, Lashmet FH, Lathrop F (1934). Ménière’s symptom complex: medical treatment. Ann Otol Rhinol Laryngol.

[19] Gantz BJ, Gidley PW, Harris JP (1999). Differential diagnosis of Meniere’s disease. Meniere’s disease.

[20] Guild SR (1927). The circulation of the endolymph. Am J Anat.

[21] Gürkov R (2017). Menière and friends. Otol Neurotol.

[22] Hallpike CS, Cairns H (1938). Observations on the pathology of Ménière’s syndrome. J Laryngol Otol.

[23] House WF (1962). Subarachnoidal shunt for drainage of endolymphatic hydrops. A preliminary report. Laryngoscope.

[24] Kiang NYS (1989) An auditory physiologist’s view of Menière’s syndrome. In: Nadol JB Jr., (ed) Meniere’s disease. Kugler Publications, The Hague, pp 13–24

[25] Kim SH, Marcus DC (2011). Regulation of sodium transport in the inner ear. Hear Res.

[26] Kimura RS, Schuknecht HF (1965). Membranous hydrops in the inner ear of the guinea pig after obliteration of the endolymphatic sac. ORL.

[27] Kitahara M, Yazawa Y, Kitahara M (1990). Secondary or idiopathic endolymphatic hydrops?. Ménière’s disease.

[28] Konishi T, Hamrick PE, Walsh PJ (1978) Ion transport in guinea pig cochlea. I. Potassium and sodium transport. Acta Otolaryngol 86(1–6):22–34 10.3109/00016487809124717 696294

[29] Loffing J (2000). Differential subcellular localization of ENaC subunits in mouse kidney in response to high- and low-Na diets. Am J Physiol Renal Physiol.

[30] Loffing J, Summa V, Zecevic M, Verrey F (2001). Mediators of aldosterone action in the renal tubule. Curr Opin Nephrol Hypertens.

[31] Lopez-Escamez JA (2015). Diagnostic criteria for Meniere’s disease. J Vestib Res.

[32] Lundquist PG, Kimura R, Wersäll J (1964). Experiments in endolymph circulation. Acta Otolaryngol.

[33] Merchant SN, Adams JC, Nadol JB (2005). Pathophysiology of Meniere’s syndrome: are symptoms caused by endolymphatic hydrops?. Otol Neurotol.

[34] Merchant SN, Nadol JB (2010). Schuknecht’s pathology of the ear.

[35] Mori N, Miyashita T, Inamoto R, Matsubara A, Mori T, Akiyama K, Hoshikawa H (2017). Ion transport its regulation in the endolymphatic sac: suggestions for clinical aspects of Meniere’s disease. Eur Arch Otorhinolaryngol.

[36] Ménière P (1861). Mémoire sur des lésions de l’oreille interne donnant lieu a des symptômes de congestion cérébrale apoplectiforme. Gaz Méd Paris.

[37] Nakashima T (2012). A perspective from magnetic resonance imaging findings of the inner ear: relationships among cerebrospinal, ocular and inner ear fluids. Auris Nasus Larynx.

[38] O’Malley JT, Burgess BJ, Jones DD, Adams JC, Merchant SN (2009). Techniques of celloidin removal from temporal bone sections. Ann Otol Rhinol Laryngol.

[39] Paparella MM, Djalilian HR (2002). Etiology, pathophysiology of symptoms, and pathogenesis of Meniere’s disease. Otolaryngol Clin North Am.

[40] Paparella MM, Goycoolea M (2016). Endolymphatic SAC enhancement surgery for MeniÃ¨re's disease an extension of conservative therapy. Annals Otol Rhinol Laryngol.

[41] Portmann G (1927). Vertigo: surgical treatment by opening the saccus endolymphaticus. Arch Otolaryngol Head Neck Surg.

[42] Pullens B, Verschuur HP, van Benthem PP (2013). Surgery for Meniere's disease. Cochrane Database Syst Rev.

[43] Pyykkö I, Nakashima T, Yoshida T, Zou J, Naganawa S (2013). Ménière’s disease: a reappraisal supported by a variable latency of symptoms and the MRI visualisation of endolymphatic hydrops. BMJ Open.

[44] Rasband WS (1997–2015) ImageJ. U.S. National Institutes of Health, Bethesda

[45] Rauch SD (2010). Clinical hints and precipitating factors in patients suffering from Meniere’s disease. Otolaryngol Clin North Am.

[46] Rauch SD, Merchant SN, Thedinger BA (1989). Meniere’s syndrome and endolymphatic hydrops. Ann Otol Rhinol Laryngol.

[47] Salt AN, Plontke SK (2010). Endolymphatic hydrops: pathophysiology and experimental models. Otolaryngol Clin North Am.

[48] Schuknecht HF, Harris JP (1999). Histopathology of Meniere’s disease. Meniere’s disease.

[49] Silverstein H, Takeda T (1976). Sodium loading of inner ear fluids. Ann Otol Rhinol Laryngol.

[50] Sood AJ, Lambert PR, Nguyen SA, Meyer TA (2014). Endolymphatic sac surgery for Meniere's disease: a systematic review and metaanalysis. Otol Neurotol.

[51] Titze J (2014). Sodium balance is not just a renal affair. Curr Opin Nephrol Hypertens.

[52] Yamakawa K (1938). Über die pathologische veränderung bei einem Ménière-Kranken. J Otorhinolaryngol Soc Jpn.

