# Supplementary figures and images for: Inner ear pathologies impair sodium-regulated ion transport in Meniere’s disease
Source: Acta Neuropathol. 2018 Nov 2;137(2):343–57. doi: 10.1007/s00401-018-1927-7 (PMC6513907; doi:10.1007/s00401-018-1927-7)

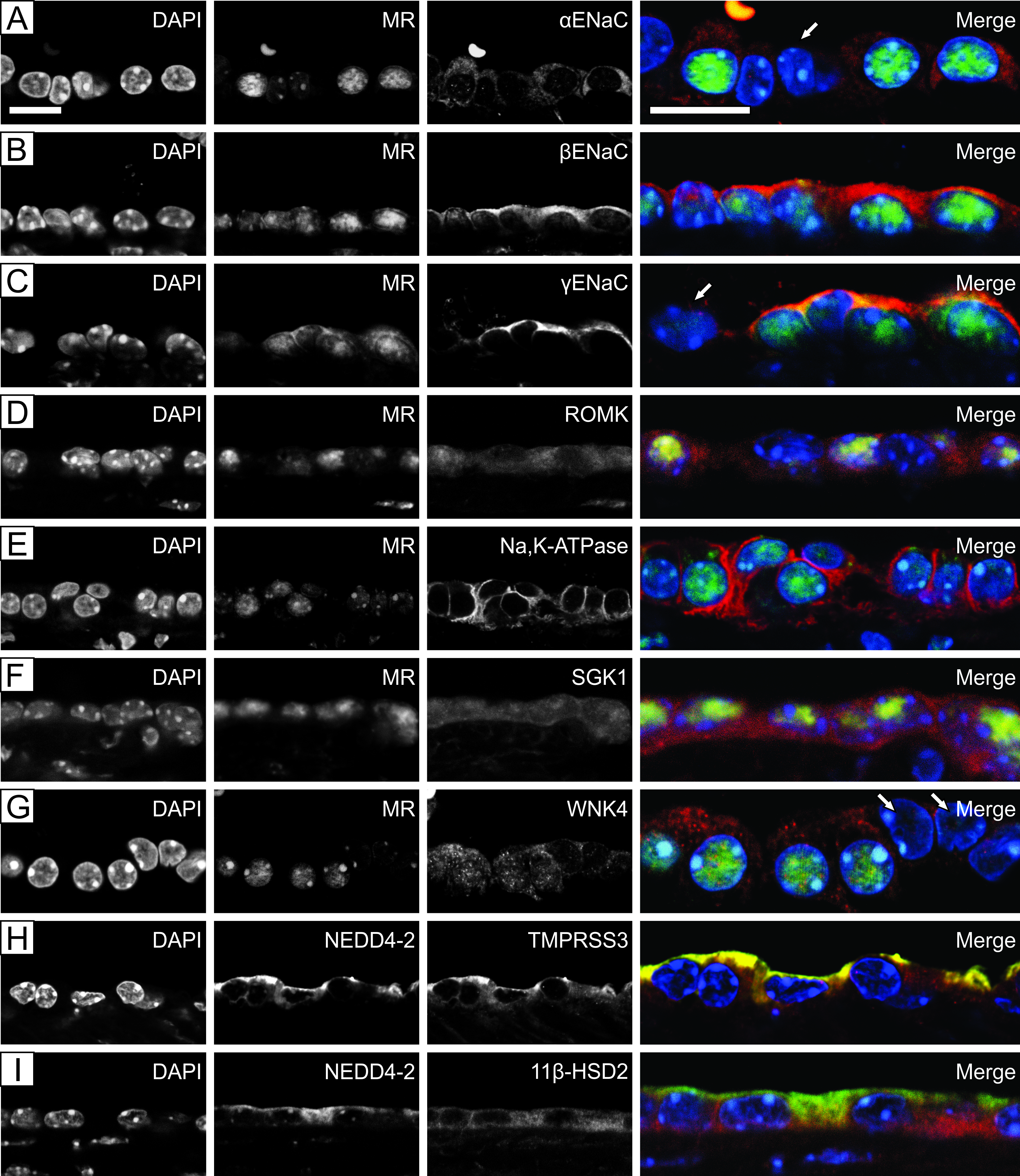

Supplement: Supplementary file 1 — Supplementary Fig. 1. Immunolocalization of ALDO-regulated Na+ transport proteins in the murine extraosseous endolymphatic sac (eES). Confocal images of double immunofluorescence labeling of the mineralocorticoid receptor (MR) and subunits α, (A), β (B), and γ (C) of the epithelial sodium channel (ENaC), the renal outer medullary potassium channel (ROMK, D), the sodium–potassium ATPase (NKA, E), serum/glucocorticoid-regulated kinase 1 (SGK1, F), and serine/threonine-protein kinase WNK4 (WNK4, G). The ALDO-regulated ion transport proteins, as well as the intracellular signaling molecules SGK1 and WNK4, are localized in MR-positive eES epithelial cells. No labeling for any of these proteins was found in MR-negative eES epithelial cells (arrows). Double labeling of the E3 ubiquitin ligase NEDD4-2 and the transmembrane protease, serine 3 (TMPRSS3, I) demonstrated cellular colocalization in eES epithelial cells. Negative control experiments in which primary antibodies were omitted did not result in any labeling in the ES epithelium (data not shown). Scale bars: 20 µm (TIFF 16655 kb) [file 401_2018_1927_MOESM1_ESM.tif]

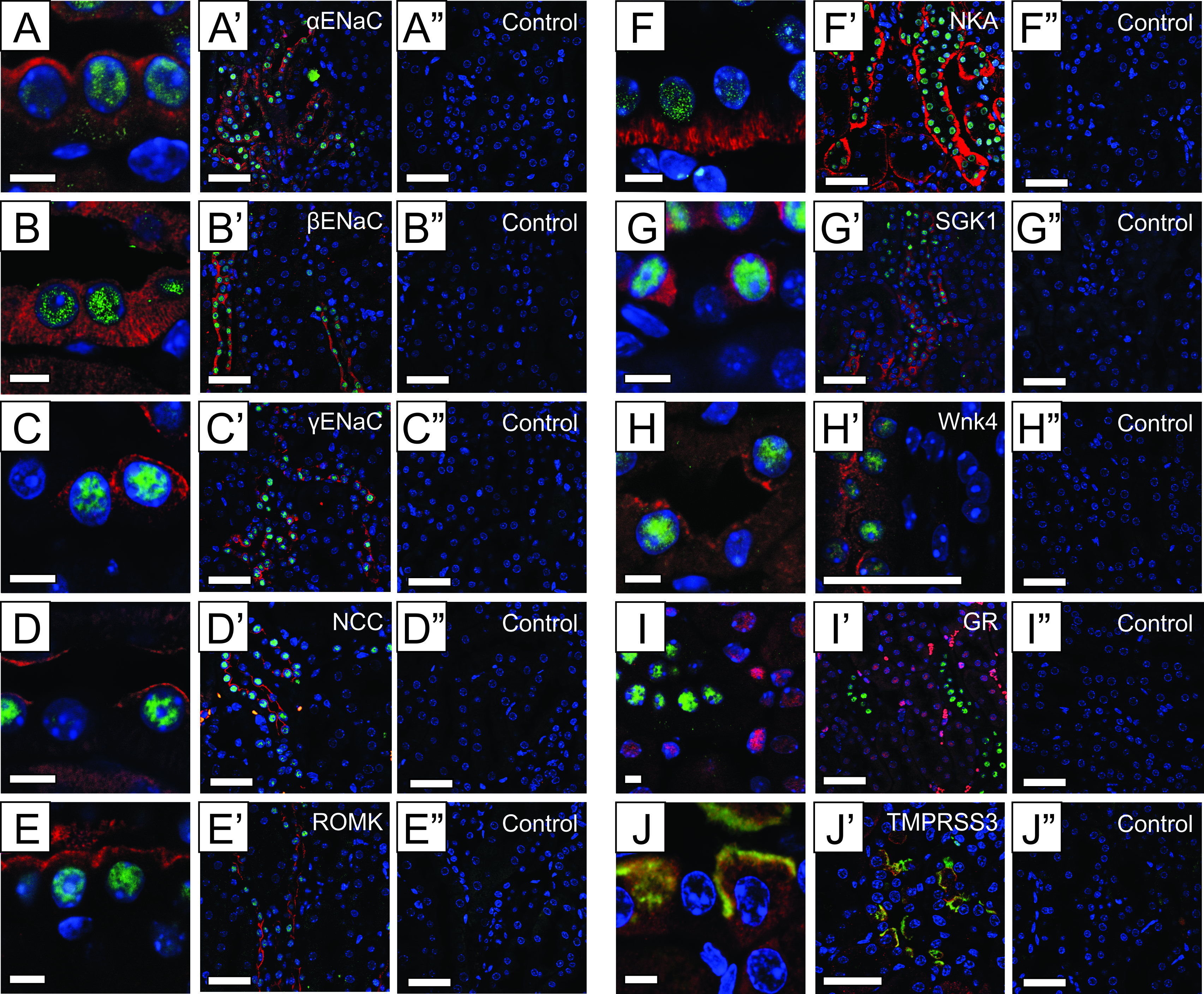

Supplement: Supplementary file 2 — Supplementary Fig. 2. Immunolocalization of ALDO-regulated Na+ transport proteins in the murine kidney. Confocal images of double immunofluorescence labeling of the mineralocorticoid receptor (MR) and subunits α, (A–A″), β (B–B″), and γ (C–C″) of the epithelial sodium channel (ENaC), the thiazide-sensitive sodium chloride cotransporter (NCC, D–D″), the renal outer medullary potassium channel (ROMK, E–E″), the sodium potassium ATPase (Na,K-ATPase, F–F″), serum/glucocorticoid-regulated kinase 1 (SGK1, G–G″), serine/threonine-protein kinase WNK4 (WNK4, H–H″), and the glucocorticoid receptor (GR, I–I″). Double labeling of the E3 ubiquitin ligase NEDD4-2 and the transmembrane protease, serine 3 (TMPRSS3, J–J″). Consistent with previous reports, all investigated proteins were localized in MR-labeled cells of the aldosterone-sensitive distal nephron (ASDN) tubular epithelium. Scale bars: (A–J), 10 µm; (A′–J′), 50 µm; (A″–J″), 50 µm (TIFF 22979 kb) [file 401_2018_1927_MOESM2_ESM.tif]

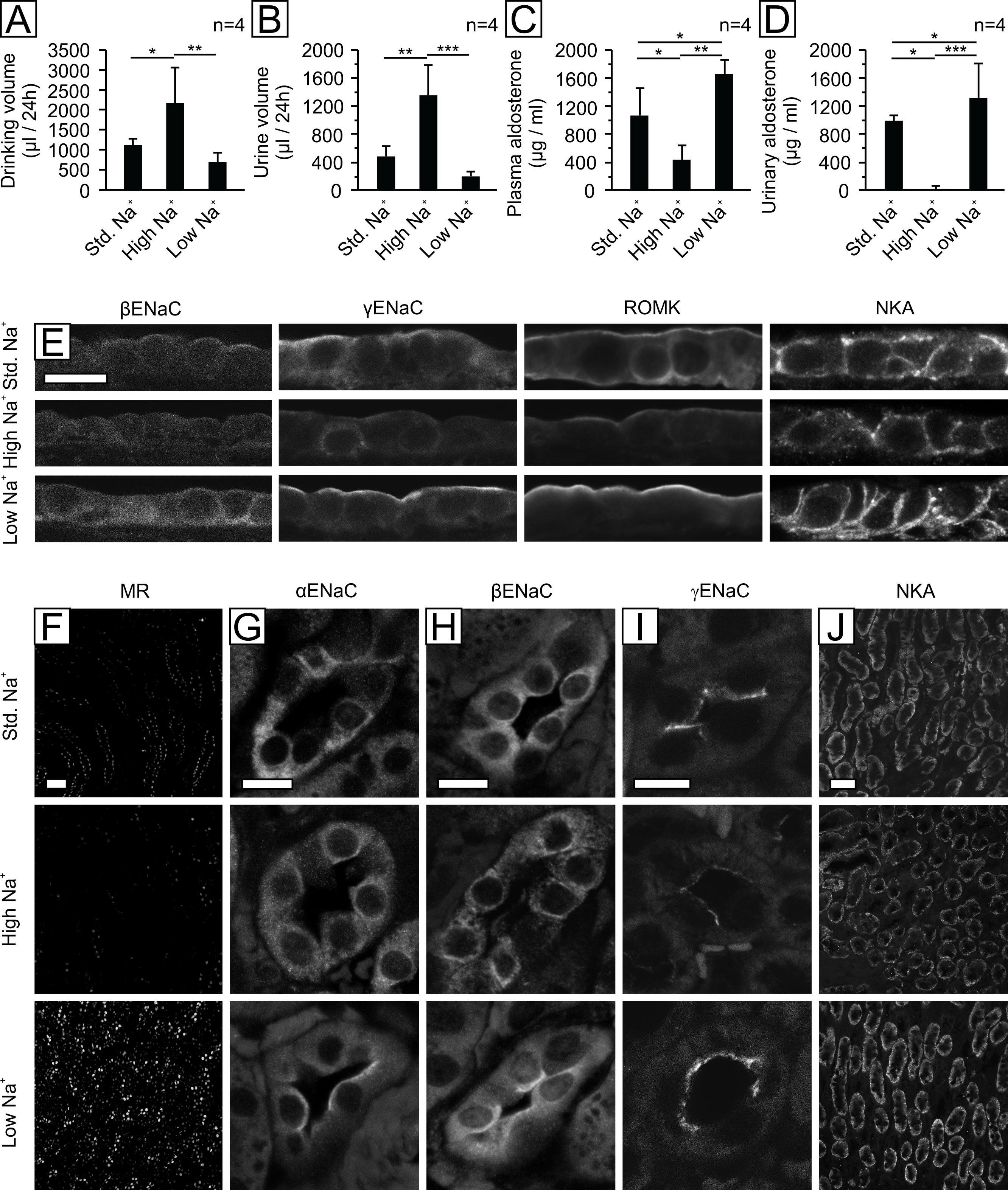

Supplement: Supplementary file 3 — Supplementary Fig. 3. (A–D) Results of metabolic balance studies on mice that were kept for 7 days on either a standard-Na+ diet (0.4%, Std. Na+), a high-Na+ diet (4.0%, High Na+), or a low-Na+ diet (0.04%, Low Na+). Diagrams showing the mean daily fluid intake (A) and output (B) per animal, as well as the mean plasma aldosterone (C) and urine aldosterone (D) levels per animal, for each of the three experimental groups. (E) Representative examples of immunolocalization patterns for βENaC, γENaC, ROMK, and NKA in the distal eES epithelium from mice fed different levels of dietary Na+ (continued from Fig. 3 (D and E)). (F–J) Representative examples of the immunolocalization patterns of MR (F); ENaC subunits α (G), β (H), γ (I); and NKA (J) in the kidneys of mice fed different levels of Na+. Statistics: (A–D) *, p ≤ 0.05; **, p ≤ 0.01; ***, p ≤ 0.001. Scale bars: (E–J), 20 µm. (TIFF 5899 kb) [file 401_2018_1927_MOESM3_ESM.tif]

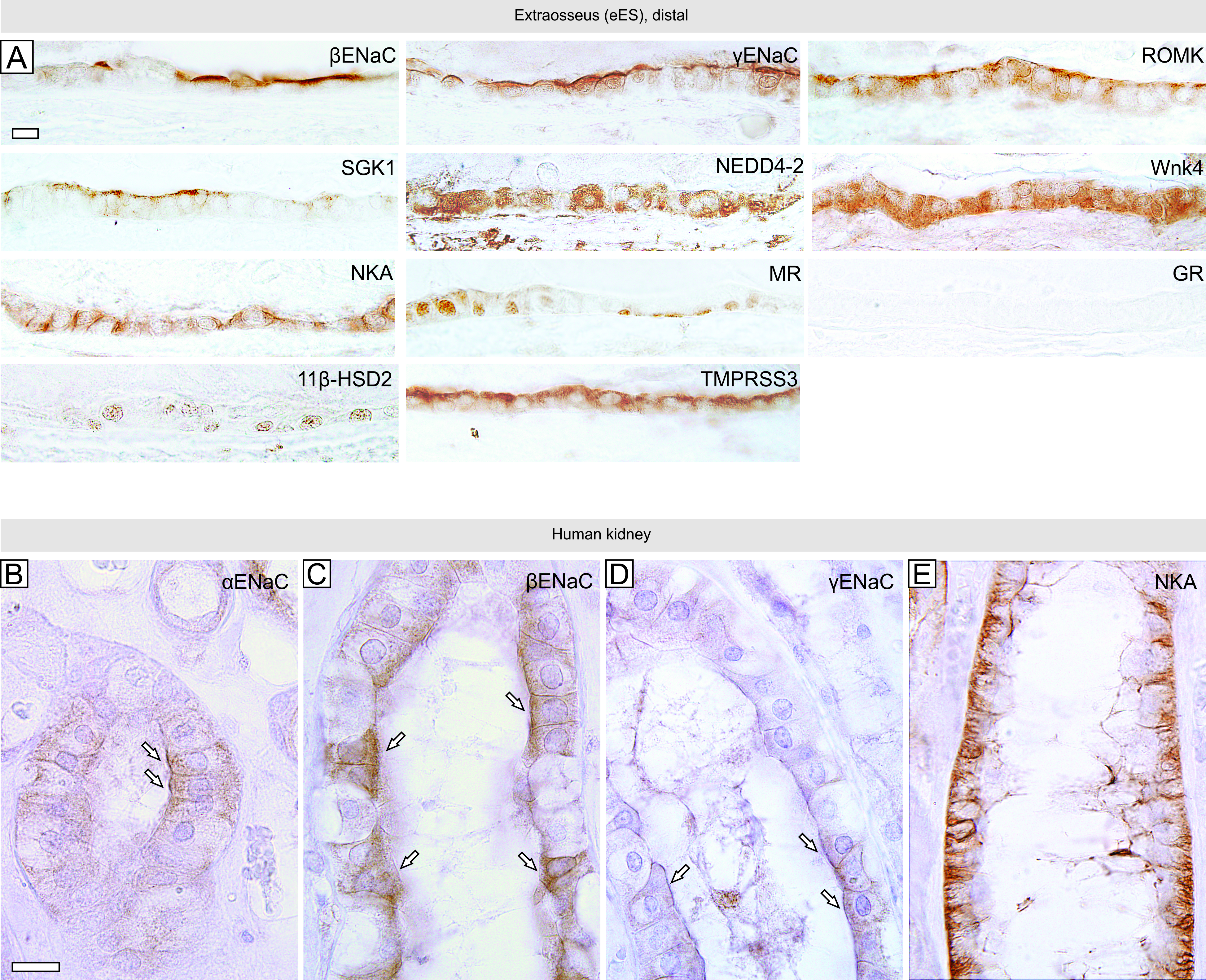

Supplement: Supplementary file 4 — Supplementary Fig. 4. Subcellular immunolocalization of ALDO-regulated Na+ transport proteins in the human distal eES and in the human kidney. (A) In the human eES, strong immunolabeling of ENaC (α, β and γ subunits) was found in the apical membranes (arrowheads) and the subapical cytoplasmic region. SGK1, NEDD4-2, WNK4 and TMPRSS3 demonstrated cytoplasmic labeling patterns (apically polarized for SGK1 and TMPRSS3). NKA labeling was seen predominantly in the lateral and basal membranes. MR and 11β-HSD2 showed a nuclear labeling pattern. No immunoreactivity for GR was found in the human ES. (B–E) In the normal human kidney (positive control tissue), immunolabeling of ENaC subunits α (B), β (C), and γ (D) was localized in apical membranes of the tubular epithelium in the cortical collecting duct (CCD). NKA was localized in basolateral epithelial membranes in the CCD (E). Immunolabeling of other proteins involved in ALDO-regulated ion transport (ROMK, NCC, MR, GR, SGK1, NEDD4-2) produced very weak labeling in the tubular epithelium (data not shown), which was presumably due to the long postmortem time of the kidney tissue sample (> 10 h). Scale bars: 20 µm (TIFF 26402 kb) [file 401_2018_1927_MOESM4_ESM.tif]

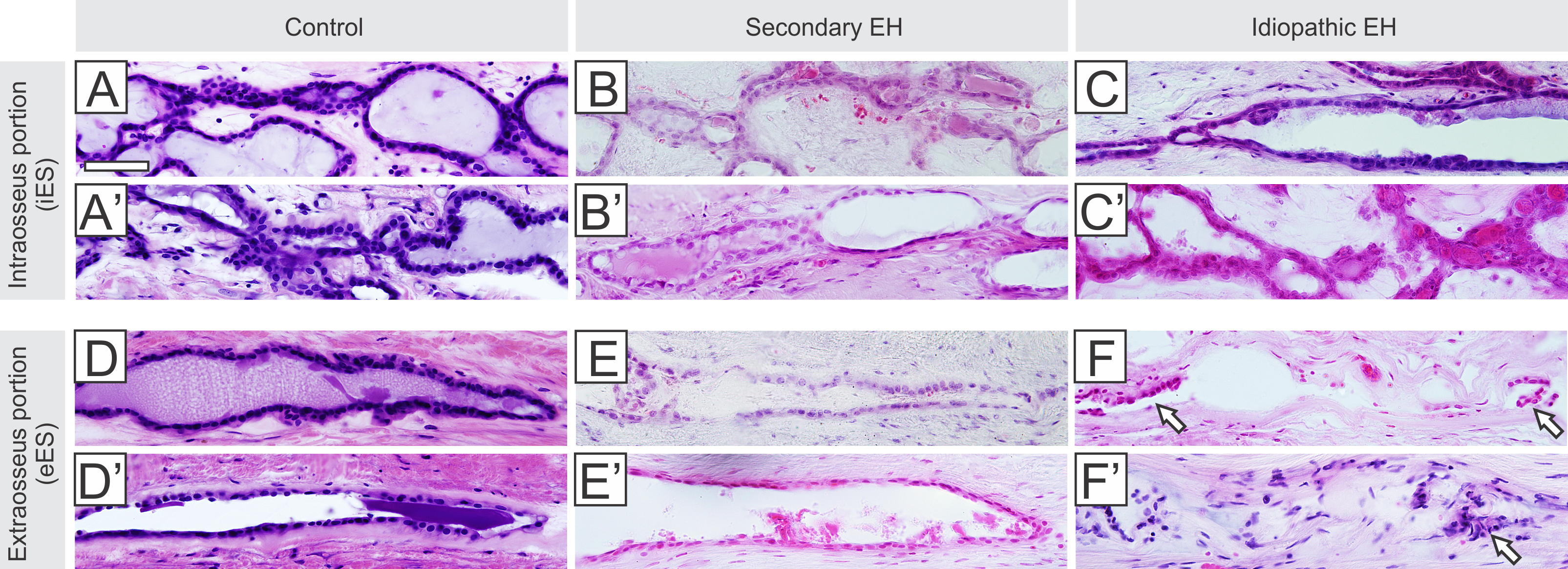

Supplement: Supplementary file 5 — Supplementary Fig. 5. Additional qualitative examples of the epithelial morphology in the iES (A–C′) and eES (D–F′) of controls, patients with secondary EH and patients with idiopathic EH. Note the epithelial damage/loss in cases with idiopathic EH (arrows in (F) and (F′) indicate remnants of eES epithelium next to areas of complete epithelial loss and fibrous replacement). Scale bars: 50 µm (TIFF 6513 kb) [file 401_2018_1927_MOESM5_ESM.tif]

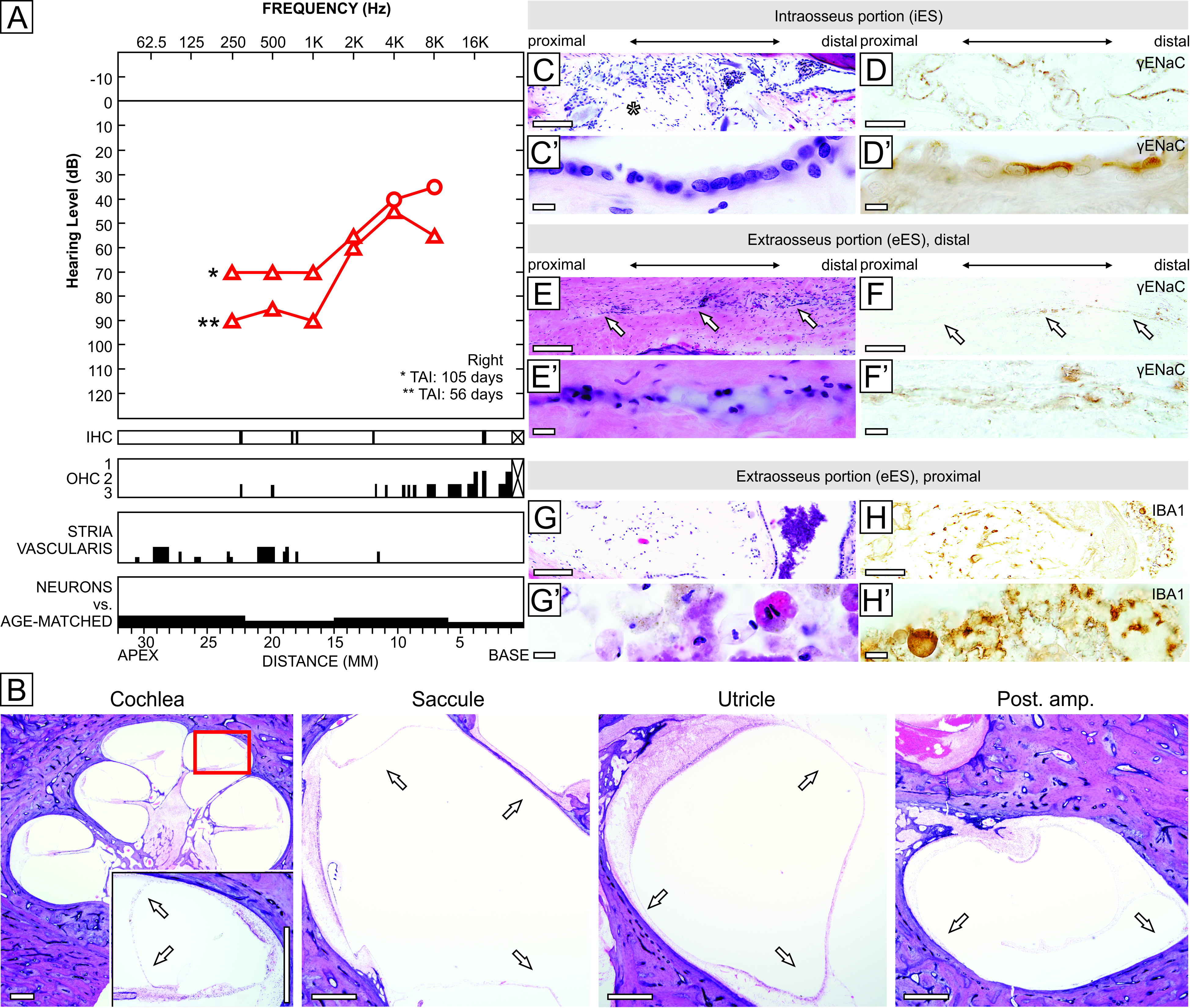

Supplement: Supplementary file 6 — Supplementary Fig. 6. A 13-year-old patient with right-sided definite MD in the early “fluctuating” disease stage. Episodes of rotational vertigo lasted for minutes, were accompanied by a feeling of pressure in the right ear, and recurred frequently over three to five consecutive days, usually in conjunction with a “flu-like” illness. (A) Audiograms at 3.5 months (*) and < 2 months (**) before death indicated severe fluctuating (or progressive) low-frequency hearing loss (TAI, time to autopsy interval). The cytocochleogram (quantification of cellular elements; black areas indicate missing cells along the tonotopic axis) for the right cochlea indicated mild scattered cellular loss, most likely caused by the 4-day postmortem time of the specimen (IHC, inner hair cells; OHC, outer hair cells). (B) In all endolymph compartments of the affected ear the epithelial membranes (arrows) were distended, indicating endolymphatic hydrops. (C–D′) Normal iES morphology on the affected side (overview in C, detail in C′) and normally weak immunolabel for γENaC (overview, D; detail, D′). (E–F′) Severe degeneration in the eES of the affected ear (overview, E; detail, E′; arrows mark the former location of the eES epithelium) and loss of immunoreactivity for ALDO-regulated proteins (overview, F; detail, F′; arrows mark the corresponding location as in (E)). (G–H′) Immune cells in the lumenal and perisaccular tissue of the iES (overview, G; detail, G′), many of which were positive for macrophage-marker IBA1 (H–H′). The clinically unaffected left inner ear showed no EH (data not shown). The histomorphology of the left eES could not be analyzed due to artifactual tissue damage caused during removal of the left temporal bone specimen. Scale bars: (B), 100 µm; (C, D, E, F, G, H), 50 µm; (C′, D′, E′, F′, G′, H′), 20 µm (TIFF 13723 kb) [file 401_2018_1927_MOESM6_ESM.tif]

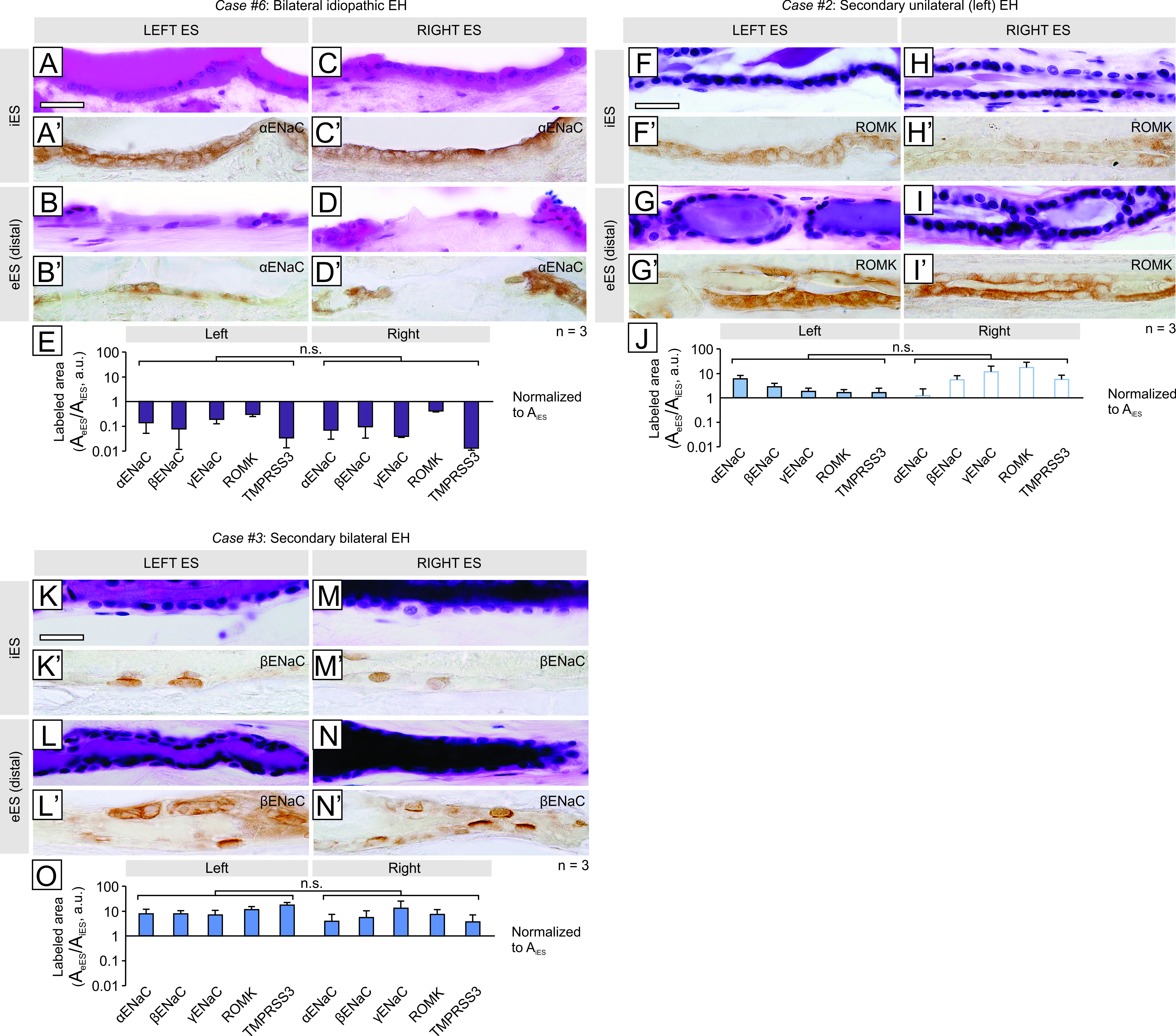

Supplement: Supplementary file 7 — Supplementary Fig. 7. Immunohistochemical labeling of ALDO-regulated proteins in human patients with idiopathic or secondary EH. (A–E) Case #6, with bilateral idiopathic EH and a clinical history of bilateral MD. The iES epithelium on both sides is intact (A, C) and exhibits normal (weak cytoplasmic) immunolabeling for αENaC (A′, C′). In contrast, the eES epithelium on both sides showed signs of severe degeneration (B, D) and very weak, nonpolarized immunolabeling patterns for αENaC (B′, D′), resulting in loss of the proximal-to-distal expression gradient (AeES/AiES ratios < 1) for ENaC (α, β, γ), ROMK and TMPRSS3 in both ESs (E). Data from (E) are also shown in Fig. 6E. (F–J) Case #2, with left-sided moderate sensorineural hearing loss (SNHL) and tinnitus after a history of labyrinthitis in the left ear. Histological analysis revealed EH, most likely secondary to the labyrinthitis, in the left inner ear. The morphology of the iES (F, H) and the eES (G, I) was intact on both sides. A normal proximal-to-distal immunolabeling gradient for all investigated Na+ transport proteins was detected in both ESs (iES (F′, H′), eES (G′, I′); AeES/AiES ratios (J)). (K–O) Case #3, with a clinical diagnosis of Cogan’s syndrome and bilateral secondary EH. Otological symptoms were not Meniere-like and included bilateral SNHL, tinnitus, and balance problems that all developed after labyrinthitis. The morphology of the iES (K, M) and the eES (L, N) was intact on both sides. A normal proximal-to-distal immunolabeling gradient for all investigated Na+ transport proteins was detected in both ESs (iES (K′, M′), eES (L′, N′); AeES/AiES ratios (O)). Statistics: (E, J, O), one-way ANOVA; n.s., not significant. Scale bars: (A–D′, F–I′, K–N′), 50 µm (TIFF 16163 kb) [file 401_2018_1927_MOESM7_ESM.tif]
