# Supplementary material for: Inner ear pathologies impair sodium-regulated ion transport in Meniere’s disease
Source: Acta Neuropathol. 2018 Nov 2;137(2):343–57. doi: 10.1007/s00401-018-1927-7 (PMC6513907; doi:10.1007/s00401-018-1927-7)
Supplement: Supplementary file 8 — Supplementary material 8 (DOCX 129 kb) [file 401_2018_1927_MOESM8_ESM.docx]

**Supplementary Table 1.** Antibodies and immunohistochemical protocols.

| **DAB immunolabeling of murine/human endolymphatic sac and kidney tissues** | | | | | | | | | | |
| --- | --- | --- | --- | --- | --- | --- | --- | --- | --- | --- |
| *Primary AB* | *Host* | *Dilution* | *Cat. #* | *Vendor/source* | *Fixative* | *HIAR* | *BT ampl.* | *Secondary AB* | | *Host* |
| Anti-αENaC | Rabbit | 1:2,000 | - | Prof. J. Loffing, Zurich, Switzerland | F/FA | Yes | Yes | Bt anti-rabbit | | Donkey |
| Anti-βENaC | Rabbit | 1:4,000 | - | Prof. J. Loffing, Zurich, Switzerland | F/FA | Yes | No | Bt anti-rabbit | | Donkey |
| Anti-γENaC | Rabbit | 1:10,000 | - | Prof. J. Loffing, Zurich, Switzerland | F/FA | Yes | No | Bt anti-rabbit | | Donkey |
| Anti-NCC | Rabbit | 1:2,000 | GTX41969 | GeneTex, Irvine, CA | F/FA | Yes | Yes | Bt anti-rabbit | | Donkey |
| Anti-ROMK | Rabbit | 1:1,000 | APC-001 | Alomone Labs, Jerusalem, Israel | F | Yes | Yes | Bt anti-rabbit | | Donkey |
| Anti-NKA | Rabbit | 1:50,000 | 31B | Dr. G. Siegel, Ann Arbor, MI | F/FA/FG | No | No | Bt anti-rabbit | | Donkey |
| Anti-MR | Mouse | 1:10,000 | rMR1-18 1D5 | DSHB, University of Iowa, Iowa City, IA | F/FA/FG/FGA | Yes | Yes | Bt anti-mouse | | Donkey |
| Anti-GR | Rabbit | 1:5,000 | M-20 | Santa Cruz, Dallas, TX | FA/FG | Yes | Yes | Bt anti-rabbit | | Donkey |
| Anti-SGK1 | Rabbit | 1:1,000 | AP7056a | Abgent, San Diego, CA | FA | Yes | Yes | Bt anti-rabbit | | Donkey |
| Anti-WNK4 | Rabbit | 1:1,000 | NB600-284 | Novus Biologicals, Littleton, CO | F | Yes | Yes | Bt anti-rabbit | | Donkey |
| Anti-NEDD4-2 | Rabbit | 1:5,000 | 21698-1-AP | Proteintech, Rosemont, IL | FA | No | Yes | Bt anti-rabbit | | Donkey |
| Anti-11β-HSD2 | Sheep | 1:1,000 | AB1296 | Chemicon Millipore | FGA | No |  | Bt anti-sheep | | Donkey |
| Anti-TMPRSS3 | Rabbit | 1:3,000 | SAB4502773 | Sigma-Aldrich (Merck), St. Louis, MO | F/FA/FG/FGA | No | Yes | Bt anti-rabbit | | Donkey |
| Anti-IBA1 | Rabbit | 1:1’000 | 019-19741 | Wako Chemicals USA Inc, Richmond, VA | F/FA/FG | No | No | Bt anti-rabbit | | Donkey |
| **Fluorescence Immunolabeling of murine endolymphatic sac and kidney tissues** | | | | | | | | | | |
| *Primary antibody* | *Host* | *Dilution* | *Cat. #* | *Vendor/source* | *Fixative* | *HIAR* | *BT amplification* | *Secondary antibody* | *Host* | *Fluorescent tag* |
| Anti-αENaC | Rabbit | 1:100 | - | Prof. J. Loffing, Zurich, Switzerland | F | Yes | No | Anti-rabbit, Alexa 568 | Donkey | - |
| Anti-βENaC | Rabbit | 1:300 | - | Prof. J. Loffing, Zurich, Switzerland | F | Yes | No | Anti-rabbit, Alexa 568 | Donkey | - |
| Anti-γENaC | Rabbit | 1:500 | - | Prof. J. Loffing, Zurich, Switzerland | F | Yes | No | Anti-rabbit, Alexa 568 | Donkey | - |
| Anti-NCC | Rabbit | 1:100 | GTX41969 | GeneTex, Irvine, CA | F | Yes | No | Anti-rabbit, Alexa 568 | Donkey | - |
| Anti-ROMK | Rabbit | 1:100 | APC-001 | Alomone Labs, Jerusalem, Israel | F | Yes | No | Anti-rabbit, Alexa 568 | Donkey | - |
| Anti-NKA | Rabbit | 1:800 | 31B | Dr. G. Siegel, Ann Arbor, MI | F | Yes | No | Anti-rabbit, Alexa 568 | Donkey | - |
| Anti-MR | Mouse | 1:200 | rMR1-18 1D5 | DSHB, University of Iowa, Iowa City, IA | F | Yes | Yes | Anti-mouse, biotinylated | Donkey | Streptavidin, Alexa Fluor 488 |
| Anti-GR | Rabbit | 1:100 | M-20 | Santa Cruz, Dallas, TX | F | Yes | No | Anti-rabbit, Alexa 568 | Donkey | - |
| Anti-SGK1 | Rabbit | 1:100 | AP7056a | Abgent, San Diego, CA | F | Yes | No | Anti-rabbit, Alexa 568 | Donkey | - |
| Anti-WNK4 | Rabbit | 1:100 | NB600-284 | Novus Biologicals, Littleton, CO | F | Yes | No | Anti-rabbit, Alexa 568 | Donkey | - |
| Anti-NEDD4-2 | Mouse | 1:300 | Ab58093 | Abcam, Cambridge, MA | FA | No | Yes | Anti-mouse, biotinylated | Donkey | Streptavidin, Alexa Fluor 488 |
| Anti-11β-HSD2 | Sheep | 1:100 | AB1296 | Chemicon Millipore | FA | No | No | Anti-sheep, Alexa 568 | Donkey |  |
| Anti-TMPRSS3 | Rabbit | 1:400 | SAB4502773 | Sigma-Aldrich (Merck), St. Louis, MO | FA | No | No | Anti-rabbit, Alexa 568 | Donkey | - |

(BT, biotinylated tyramine; F, 10 % neutral buffered formalin; FA, F + 1 % glacial acetic acid; FG, F + 0.2 % glutaraldehyde; FGA, FA + 0.2 % glutaraldehyde; HIAR, heat-induced antigen retrieval).
