# Supplementary material for: Inner ear pathologies impair sodium-regulated ion transport in Meniere’s disease
Source: Acta Neuropathol. 2018 Nov 2;137(2):343–57. doi: 10.1007/s00401-018-1927-7 (PMC6513907; doi:10.1007/s00401-018-1927-7)
Supplement: Supplementary file 9 — Supplementary material 9 (DOCX 51 kb) [file 401_2018_1927_MOESM9_ESM.docx]

**Supplementary Table 2**. Clinical diagnoses that are known to be associated with EH in patients who were classified as having secondary EH.

| **Diagnosis** | **# of cases** |
| --- | --- |
| Congenital hydrocephalus | 2 |
| Hereditary SNHL | 5 |
| Labyrinthitis | 15 |
| Neoplastic disease affecting the temporal bone | 11 |
| Acquired otosyphilis | 3 |
| Inner ear autoimmune disease | 2 |
| Otosclerosis | 9 |
| Trauma (fracture) of the temporal bone | 1 |
| Cogan's syndrome | 2 |
| Sudden SNHL | 5 |
| CI implantation | 1 |
| Cisplatin therapy | 2 |

(SNHL, sensorineural hearing loss; CI, cochlear implant).
