# Supplementary material for: Inner ear pathologies impair sodium-regulated ion transport in Meniere’s disease
Source: Acta Neuropathol. 2018 Nov 2;137(2):343–57. doi: 10.1007/s00401-018-1927-7 (PMC6513907; doi:10.1007/s00401-018-1927-7)
Supplement: Supplementary file 10 — Supplementary material 10 (DOCX 56 kb) [file 401_2018_1927_MOESM10_ESM.docx]

**Supplementary Table 3.** Data on human temporal bone specimens from the MEEI collection that were reviewed for the histopathological analysis.

|  | # of specimens (%) |
| --- | --- |
| *Key word: endolymphatic hydrops, total* | 224 (100) ^A^ |
| - Excluded   (Reasons: vertical section plane, eES not sectioned, artifactual damage of the eES) | 114 (50.9) |
| - Included | 110 (49.1) |
| - - From females | 62 (56.4) ^B^ |
| - - From males | 48 (43.6) ^B^ |
| - - Right specimen | 59 (53.6) ^B^ |
| - - Left specimen | 51 (46.4) ^B^ |
| - - Idiopathic EH | 42 (38.2) ^B^ |
| - - - Definite MD | 38 (90.5) ^C^ |
| - - - Non-MD otol. sympt. | 4 (9.5) ^C^ |
| - - - No otological symptoms | 0 (0) ^C^ |
| - - Secondary EH | 58 (52.7) ^B^ |
| - - - Meniere’s syndrome | 6 (10.3) ^D^ |
| - - - Non-Meniere’s otol. sympt. | 48 (82.8) ^D^ |
| - - - No otological symptoms | 4 (6.9) ^D^ |
| Normal controls (no history of otological disease) | 10 |

(^A^, not including 10 control cases; ^B^, % of included cases; ^C^, % of cases with idiopathic EH; ^D^, % of cases with secondary EH).
