# Supplementary material for: Inner ear pathologies impair sodium-regulated ion transport in Meniere’s disease
Source: Acta Neuropathol. 2018 Nov 2;137(2):343–57. doi: 10.1007/s00401-018-1927-7 (PMC6513907; doi:10.1007/s00401-018-1927-7)
Supplement: Supplementary file 11 — Supplementary material 11 (DOCX 226 kb) [file 401_2018_1927_MOESM11_ESM.docx]

**Supplementary Table 4.** Histopathological evaluation scale for the epithelium in the human iES and eES.

|  | **I** | | | | | **II** | | | | | **III** | | | | |
| --- | --- | --- | --- | --- | --- | --- | --- | --- | --- | --- | --- | --- | --- | --- | --- |
|  | **Epithelial integrity** | **Points** | | **Example** | | **Epithelial cell morphology** | | **Points** | **Example** | | **Nuclear morphology** | | **Points** | | **Example** |
|  | Intact | 1 | | 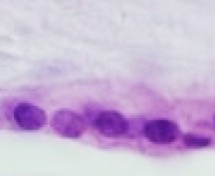 | | Columnar/ cuboidal | | 1 | 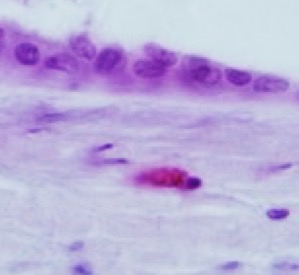 | | Round/oval | | 1 | | 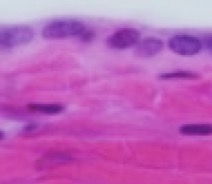 |
|  | Disarranged/ discontinuous | 2 | | 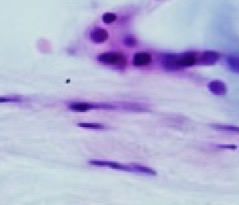 | | Squamous/ polymorphous | | 2 | 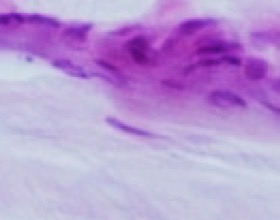 | | Irregular shaped | | 2 | | 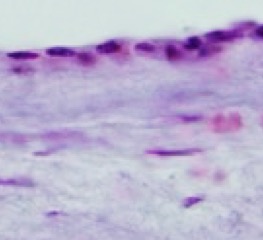 |
|  | Absent | 3 | | 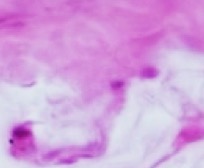 | | Absent | | 3 | 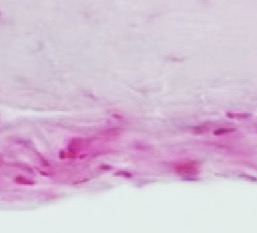 | | Pyknotic/ absent | | 3 | | 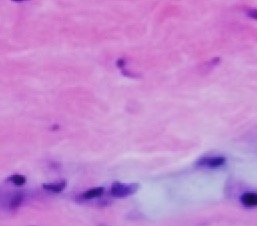 |
| **Total score**  **(I+II+III)** | **3** | | **4** | | **5** | | **6** | | | **7** | | **8** | | **9** | |
| **Rating** | **+ + +** | | **+ +** | | **+** | | **0** | | | **-** | | **- -** | | **- - -** | |

For each ES (including the iES and eES), the ratings for categories I–III were added together, resulting in a total score (3–9) and the corresponding epithelial integrity rating (+++ to ---).
