# Supplementary material for: Inner ear pathologies impair sodium-regulated ion transport in Meniere’s disease
Source: Acta Neuropathol. 2018 Nov 2;137(2):343–57. doi: 10.1007/s00401-018-1927-7 (PMC6513907; doi:10.1007/s00401-018-1927-7)
Supplement: Supplementary file 12 — Supplementary material 12 (DOCX 48 kb) [file 401_2018_1927_MOESM12_ESM.docx]

**Supplementary Table 5.** Cardiovascular/hematological diagnoses in patients with idiopathic EH.

| **Diagnosis** | **# of cases** |
| --- | --- |
| Atherosclerosis | 1 |
| Cardiac arrythmia | 1 |
| Cardiac valvulopathy | 1 |
| Cerebrovascular accidents | 3 |
| Congenital heart disease | 3 |
| Coronary artery disease | 3 |
| Hypertension | 4 |
| Myelogenous leukemia | 1 |
| Polycythemia vera | 1 |

Some patients had multiple diagnoses. Cardiovascular/hematological diagnoses were considered only when they were made prior to the onset of clinical MD.
